# A multi‐level approach reveals key physiological and molecular traits in the response of two rice genotypes subjected to water deficit at the reproductive stage

**DOI:** 10.1002/pei3.10121

**Published:** 2023-09-15

**Authors:** Bénédicte Favreau, Camille Gaal, Isabela Pereira de Lima, Gaétan Droc, Sandrine Roques, Armel Sotillo, Florence Guérard, Valérie Cantonny, Bertrand Gakière, Julie Leclercq, Tanguy Lafarge, Marcel de Raissac

**Affiliations:** ^1^ CIRAD, UMR AGAP Institut Montpellier France; ^2^ UMR AGAP Institut, Univ Montpellier, CIRAD, INRAE, Institut Agro Montpellier France; ^3^ BASF Rio Verde/GO—Soybean Breeding Rio Verde Brazil; ^4^ Plateforme Métabolisme‐Métabolome Institute of Plant Sciences Paris‐Saclay (IPS2), Université Paris‐Saclay, National Committee of Scientific Research (CNRS), National Institute for Research for Agriculture, Food and Environment (INRAE), Université d'Evry, Université de Paris Gif‐sur‐Yvette France

**Keywords:** development, gene networks, rice, transcriptomics, water deficit

## Abstract

Rice is more vulnerable to drought than maize, wheat, and sorghum because its water requirements remain high throughout the rice life cycle. The effects of drought vary depending on the timing, intensity, and duration of the events, as well as on the rice genotype and developmental stage. It can affect all levels of organization, from genes to the cells, tissues, and/or organs. In this study, a moderate water deficit was applied to two contrasting rice genotypes, IAC 25 and CIRAD 409, during their reproductive stage. Multi‐level transcriptomic, metabolomic, physiological, and morphological analyses were performed to investigate the complex traits involved in their response to drought. Weighted gene network correlation analysis was used to identify the specific molecular mechanisms regulated by each genotype, and the correlations between gene networks and phenotypic traits. A holistic analysis of all the data provided a deeper understanding of the specific mechanisms regulated by each genotype, and enabled the identification of gene markers. Under non‐limiting water conditions, CIRAD 409 had a denser shoot, but shoot growth was slower despite better photosynthetic performance. Under water deficit, CIRAD 409 was weakly affected regardless of the plant level analyzed. In contrast, IAC 25 had reduced growth and reproductive development. It regulated transcriptomic and metabolic activities at a high level, and activated a complex gene regulatory network involved in growth‐limiting processes. By comparing two contrasting genotypes, the present study identified the regulation of some fundamental processes and gene markers, that drive rice development, and influence its response to water deficit, in particular, the importance of the biosynthetic and regulatory pathways for cell wall metabolism. These key processes determine the biological and mechanical properties of the cell wall and thus influence plant development, organ expansion, and turgor maintenance under water deficit. Our results also question the genericity of the antagonism between morphogenesis and organogenesis observed in the two genotypes.

## Graphical abstract

Two rice genotypes were subjected to a moderate water deficit applied during the reproductive phase. Multi‐level transcriptomic, metabolomic, physiological, and morphological analyses were performed to investigate the complex traits involved in their response to drought. A holistic analysis of these data allowed us to identify, for each genotype, underlying mechanisms participating in growth regulation under water stress.

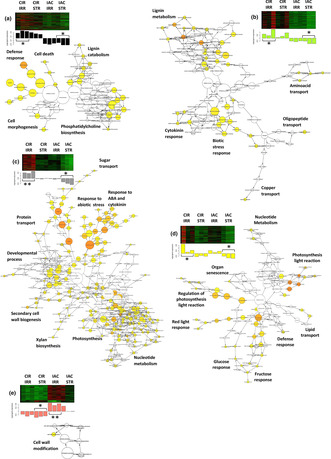

## 1 INTRODUCTION

Rainfed rice is more susceptible to drought than other cereal crops due to its semi‐aquatic origin and high‐water demand throughout its life cycle (Kumar et al., 2008). Rice is subject to stress as soon as available soil water drops below 70% of its maximum value, whereas for most crops, the threshold is around 30% (Lilley & Fukai, 1994). Drought is therefore the most important constraint to rice cultivation. It limits nutrient uptake, causes dehydration, and reduces yield in rainfed areas (Choudhury et al., 2017; Kumar et al., 2014; Price et al., 2002; Serraj et al., 2009). Plants are even more affected when drought occurs during the reproductive stage, as there is a direct impact on grain yield (Barnabas et al., 2008; Fahad et al., 2017; Fischer et al., 2003; He & Serraj, 2012; Namuco & Otoole, 1986).

Growth is defined as an irreversible increase in the number of cells, biomass, plant volume, and a combination of all these processes. Water deficit can affect the plant at different levels, which may ultimately affect growth (Hilty et al., 2021). The first processes to be affected when water potential decreases are leaf expansion and stem elongation, followed by photosynthesis and transpiration (Boyer, 1970; Fischer et al., 2003). The first effect is a reduction in the surface area of the last four leaves that provide the carbohydrates needed for grain filling after anthesis. Another consequence is a slowdown in the leaf emergence rate and associated phyllochron lengthening, leading to a delay in anthesis (Lafitte et al., 2003). As stem elongation is also reduced, panicle exertion is incomplete or, in the case of severe stress, may even inhibited along with flowering (Cruz & O'Toole, 1984; Ekanayake et al., 1989; He & Serraj, 2012; O'Toole & Namuco, 1983). Finally, when water deficit occurs during the reproductive stage, there may be a reduction in spikelet formation and abnormal chromosomal behavior during meiosis in the microspore mother cells (Namuco & O'Toole, 1986), resulting in pollen sterility (Sheoran & Saini, 1996). By reducing expansion prior to photosynthesis, water deficit leads to the accumulation of non‐structural sugars in many other crops including rice (Cabuslay et al., 2002; Franck et al., 2006; Luquet et al., 2006, 2008; Rebolledo et al., 2012). Along with phenotypic changes, drought regulates several molecular mechanisms, including signal transduction, protein metabolism, synthesis of compatible compounds and of plant hormones, and carbohydrate metabolism (Harb et al., 2010; Lata et al., 2015; Yamaguchi‐Shinozaki & Shinozaki, 2006). To regulate these processes, plants rely on gene networks that orchestrate dynamic changes and metabolic activity, and that in turn, allow the plant to rapidly adapt to environmental constraints (Wilkins et al., 2016).

Plant responses to environmental constraints result from complex interactions between morphological, physiological, biochemical, and molecular features. Despite the considerable impact of drought on the reproductive development of rice, plant response remains to be elucidated, in particular at the molecular and metabolic levels (Ganie & Ahammed, 2021). A multi‐level approach is consequently the best way to acquire deeper insights into the regulation of the biological mechanisms involved (Bardini et al., 2017; Hilty et al., 2021). Plant responses also depend on their genotype, physiology, and developmental stage. Studying the effect of water deficit on plants at the same developmental stage is challenging because it is extremely difficult to apply exactly the same limiting water supply to genotypes that differ in their development and growth rates. While some studies have evaluated the omics response of water‐stressed rice at the reproductive stage (Gour et al., 2021; Lenka et al., 2011; Liang et al., 2021; Wilkins et al., 2016; Yoo et al., 2017; Zhang et al., 2016), few applied the moderate level of stress that more closely matches agronomic conditions (Barnaby et al., 2019; Liang et al., 2021; Plessis et al., 2015; Torres & Henry, 2018). It is indispensable to understand the complex biological mechanisms and the polygenic traits that regulate developmental processes under moderate stress.

In the present study, a multi‐level approach was applied to fine‐scale the response of two rice genotypes under moderate drought conditions applied during the early reproductive stage. The two genotypes were selected based on the results obtained in a previous experiment within which six genotypes were planted using similar conditions in two different environments: a greenhouse in Goiania (Brazil) and a growth chamber in Montpellier (France). The six genotypes were subjected to a moderate water deficit applied during their reproductive stage that affected the same basic processes at the same intensity and with the same duration (Pereira de Lima et al., 2021). The contrasted response of CIRAD 409, the more tolerant genotype, and IAC 25, the more sensitive genotype, was highlighted at both sites. In the present study, the multi‐level morphological, physiological, biochemical, metabolomic, and transcriptomic responses of CIRAD 409 and IAC 25 were evaluated under non‐limiting conditions (with irrigation) and under limiting conditions (with a moderate water deficit), using the strategy described in Figure S1. The data were analyzed holistically, thereby revealing underlying biological mechanisms and identifying key genes involved in developmental process, that otherwise would not have been discovered. The results of the present study clearly demonstrate the advantages of applying a multi‐level and holistic approach to unravel the complex mechanisms that regulate plant development under stress.

## 2 MATERIALS AND METHODS

### 2.1 Plant material and experimental design

Two upland rice genotypes were used for this study, CIRAD 409 (CIR) and IAC 25 (IAC), that were selected for their contrasted response to water deficit during a previous experiment (Pereira de Lima et al., 2021). The present experiment was conducted in a growth chamber with three plants per genotype and per treatment (with irrigation vs. a moderate water deficit) following a completely randomized design. The growth chamber parameters were 12 h/12 h photoperiod, 28°C/20°C day/night temperatures, 65%/90% day/night relative air humidity (average daily VPD of 1.4 kPa), mean incident radiation of 400 μM/m^2^/s at the last ligulated leaf. Pots were filled with 1470 g of a moist commercial loam soil (TRefRiz Cirad 2) designed by Cirad specifically for rice cropping. When the pots were filled, soil samples were systematically collected to determine the humidity and total dry weight in each pot. The fraction of transpirable soil water (FTSW) was then calculated using the following formula (Sinclair & Ludlow, 1986):

$$\text{FTSW}=\frac{\left(\text{Humid soil weight}-\text{Soil weight}\;\mathrm{at}\;\text{wilting point}\right)}{\text{Soil weight}\;\mathrm{at}\;\text{field capacity}-\text{Soil weight}\;\mathrm{at}\;\text{wilting point}}.$$

The water available in each pot was thus monitored simply by weighing. The water retention properties of the soil determined from pressure/volume curves, were 3.5 g H_2_O/g dry weight (DW) at field capacity (FC), and 0.35 g H_2_O/g DW at wilting point (WP). The soil in the pots was covered with polystyrene balls to prevent direct evaporation from the soil, and plant transpiration rate was monitored.

The panicle initiation (PI) date of each genotype was identified in the previous experiment performed in the same controlled conditions (Pereira de Lima et al., 2021). Pots were irrigated at 80% of field capacity (FTSW = 0.8 = 2.87 g/g), from planting to the onset of the differential water treatments, that started 5 days after panicle initiation (PI). At this date, which differed in the two genotypes, two treatments were applied: (1) “IRR,” the control treatment with maintenance of full irrigation; (2) “STR,” water deficit stress with an initial 18‐day period with no irrigation until FTSW reached 0.4 (1.66 g/g), followed by an 11‐day period with pot adjustment to FTSW = 0.4, three times a week (Figure S2). To avoid the effects of spatial heterogeneity, the pots were rotated after irrigation, three times a week. PI and heading in IAC occurred 4 days later than in CIR. For each genotype, the experiment ended at heading in irrigated plants. The plants were then collected and dissected. The kinetic, duration and intensity of the water deficit were thus identical, and were managed at the same physiological stages in the two genotypes despite their physiological differences.

### 2.2 Measurement of phenotypic traits

Herein, the term ‘phenotypic traits’ refer to all phenological, morphological, and physiological traits as well as sugar content, used to characterize the whole plant or one of its organs (Table S1). Phenotypic traits were measured throughout the experiment using non‐destructive monitoring, or at the end of the experiment using destructive measurements. Measurements were taken on three plants per genotype and per treatment, and additional samples were collected for further analysis. Non‐destructive monitoring included the Haun Index (HI), a visual quantification of plant development (Haun, 1973). The HI was used to calculate the phyllochron, i.e., the interval between the emergence of two consecutive leaves on the main stem. The phyllochron was calculated from the beginning of the application of stress to flag leaf ligulation (Phyllo_Repr). The CO_2_ assimilation rate (An), transpiration rate (Tr), and internal CO_2_ content (Ci) were measured on the flag leaf of the main stem at the end of the experiment between 9:00 a.m. and 11:00 a.m. The chamber was illuminated at 8:00 a.m. The net assimilation rate was measured with a Walz GFS 3000 (Heinz Walz GmbH) in the following chamber conditions: large exchange area cuvette of 8 cm^2^, light intensity PAR TOP of 1500 μmol m^−2^ s^−1^; flow rate of 850 μmol/min; impeller of 8; relative humidity of 65%; chamber temperature of 28°C; CO_2_ control at 400 ppm. An/Tr ratio was calculated (InstEndWUE), along with the assimilation rate per unit of chlorophyll (An/Spad). Cumulative water consumption was calculated as the sum of daily transpiration measured during pot irrigation throughout the period with differentiated water treatment. During the same period, the cumulative water use efficiency (CumWUE) was defined as the ratio of biomass accumulation to water consumption, the former was estimated using the method described in (Pereira de Lima et al., 2021). When panicles emerged in the fully irrigated treatment, each irrigated and stressed plant was dissected. The tillers were counted (Tiller_N°), and the main tiller was separated from the others. The main tiller was weighed (Tiller_Biom) and its height measured from the base to the flag leaf ligule (Plant_Height). Then the length, width and diameter of the following organs were measured: panicle (Pan_Length), peduncle (Ped_Length, Ped_Diam), flag leaf (FlagLeaf_Length, FlagLeaf_Width), first leaf below (F‐1_Length, F‐1_Width), successive internodes from the top to the base (IN1_Length, IN1_Diam, IN2_Length, IN2_Diam, IN3_Length, IN3_Diam). The panicle was scanned and the total branch length (TotBranch_Length) was determined using P‐TRAP software. The length (INTot_Length) and weight (INTot_Biom) of the internodes were recorded. The number of branches was determined by summing the primary and secondary branches (TotBranch_N°), and the number of spikelets was counted (Spiklt_N°). After the main stem was dissected, the following organs were weighed: panicle (Pan_Biom), blade (Blade_Biom), and sheath biomass (Sheat_Biom). The sum of all organs of the main tiller was calculated (MainTill_Biom). The cumulative weight of all the organs of the main tiller and all the other tillers represents the shoot biomass (Shoot_Biom). The total leaf area of the plant (TotLeaf_Area) was measured, i.e., the leaf area of all the tillers and of the main stem. Finally, a morpho‐physiological trait, specific leaf area (SLA), was calculated as the ratio of leaf area to leaf biomass. For sugar analysis, the flag leaf blade (FL) and internodes 1 and 2 (IN1, IN2) were sampled early in the morning, immediately frozen in liquid nitrogen and then lyophilized for 72 h. Samples were then ground in a Retsch MM400 ball mill (particles <50 μm) and the resulting powders were stored at −80°C until analysis. Glucose, fructose, sucrose, and starch contents were determined according to the method described in Luquet et al. (2006). Results are expressed in mg g^−1^, as soluble sugars per unit of dry matter for hexose (glucose + fructose) and sucrose, in the flag leaf blade (FL) and internodes 1 and 2 (IN1, IN2). A partial least squares discriminant analysis (PLS–DA) was performed using the mixOmics R package to assess the quality of dataset and to explore its underlying structure (Rohart et al., 2017). Comparison of variance between groups was calculated using a non‐parametric Kruskall–Wallis test (Rstatix R package), as recommended when the sample size is less than 30. Significant differences were detected (if *p*‐value < .05) between genotypes, treatments, and/or genotype × treatment. To increase confidence in our analyses, the magnitude of the difference between groups was measured by calculating the effect size eta squared, based on the H statistic (eta^2^[H]). The difference between groups was considered to be small if 0.01 > eta^2^[H] < 0.06, moderate if 0.06 > eta^2^[H] < 0.14, and large if eta^2^[H] ≥ 0.14.

### 2.3 RNA sequencing analysis

#### 2.3.1 Sampling and sequencing

Transcriptomic analysis was performed on the same frozen powder made of internode 1 as that collected for sugar analysis. This internode, which taken from the first primary tiller and located just below the peduncle, was selected for this analysis because it was elongating at the sampling date (Counce et al., 2000). Total RNA was extracted from 200 mg of each sample using the TRIzol protocol (Chomczynski & Sacchi, 2006). The quantity and quality of total RNA were determined using the Agilent 2200 TapeStation system. Samples with an RNA integrity number (RIN) value >8 were deemed acceptable and sequenced using the Illumina TruSeq RNA protocol (Illumina Inc.). Sequencing was conducted as paired‐end reads (length 150 bp) in a single lane of a flow cell on the Illumina HiSeqTM 3000, at the INRAE Genotoul platform. Prior to sequence assembly, read quality was checked using FastQC (v 0.11.3), before and after adapter removal using Cutadapt (Martin, 2011). Reads were then trimmed if the length <35 bp and PHRED score <30. Good quality mRNA‐seq reads were aligned to the *Oryza sativa* spp. *japonica* reference genome using Hisat2 (v2.1.0) (Kim et al., 2015). The reads were annotated using the MSU Rice Genome Annotation Project database (http://rice.uga.edu/). Gene expression levels (RPKM) were estimated using Edge R (v 3.20.1) (Robinson et al., 2009). Fastq files of the sequenced libraries were deposited in the publicly accessible NCBI Sequence Read Archive (SRA) under accession numbers: SAMN27520494, SAMN27520495.

#### 2.3.2 Gene selection by pairwise comparison and gene network analysis

Genes expressed according to the water treatment and/or genotype were selected using both pairwise comparison and network analysis following the strategy described in Favreau et al., 2019. First, all the genes whose expression level changed with the genotype, treatment, or with the interaction between the two factors were selected using the LRT test following the multifactorial design protocol in the DESeq2 R package described in http://bioconductor.org/packages/release/bioc/html/DESeq.html (Love et al., 2014). All the treatments were tested simultaneously according to the multifactorial model: Genotype + Treatment + Genotype × Treatment. All significant genes with false discovery rate corrected *p*‐values < .01 threshold were selected to build the gene set, hereafter named Multifactor. To assess the quality of the gene set, and to explore its underlying structure, PLS–DA was performed as described above. Next, differentially expressed genes (DEGs) and networks were extracted from the Multifactor gene set. Significant DEGs were identified for each water‐deficient versus irrigated genotype by pairwise comparison using the Wald test (DESeq2 R package), and were selected if they had a false discovery rate corrected *p*‐values < .01. Gene network analysis was performed using the Weighted Gene Co‐expression Network Analysis (WGCNA R package) (Langfelder et al., 2013), as described at https://labs.genetics.ucla.edu/horvath/CoexpressionNetwork/Rpackages/WGCNA/Tutorials/. Briefly, counts of the Multifactor gene set, normalized by their relative standard deviation (RSD), were used to build groups of highly correlated genes, and clustered in modules based on their dissimilarity using the following settings: power = 18, minModuleSize = 90, MEDissThres = 0.1. A histogram was generated for each network to show the mean level of gene expression for each sample, and the significance of the gene expression for the genotype and treatment effect (*p*‐value < .05). Based on mean gene overexpression levels, networks that represented the response of each genotype to irrigated or water‐limited conditions were selected for further analysis. Correlations were computed between these networks and treatments, on the one hand, and with phenotypic variables, on the other hand. Phenotypic variables that were highly correlated with one another were removed. Correlation values were considered significant if the *p*‐value was <.05. Finally, hub genes were selected for the phenotypic variables the most highly correlated with the networks of interest. According to the WGCNA procedure, only genes with the highest significance for the correlated phenotypic variable (|GS| > 0.90), and membership (MM > 0.90) were considered. Gene significance (GS) is the log^10^ transformation of the *p*‐value in the linear regression between gene expression and phenotypic variables. Module membership (MM) is the correlation of the module eigengenes (ME) with the gene expression profile for each gene in the module. The MM reflects the connectivity, or the sum of the connection strengths with the other genes in the network. The higher the MM value of a gene, the closer its connection to the other genes in the given module.

#### 2.3.3 Functional analysis of DEGs and networks

Gene ontology enrichment of biological processes was performed for the DEGs and network genes using the BiNGO plugin (Cytoscape© software), based on *Oryza sativa* annotation (Lopes et al., 2010; Maere et al., 2005). Default parameters were used. Briefly, over‐representation of biological processes was assessed. The hypergeometric test was used as the statistical test, followed by multiple testing correction using the significant false discovery rate correction at *p*‐value < .05. Map enrichment was designed to summarize and visualize enriched biological processes. Gene ontology (GO) enrichment generated by BiNGO was used to implement the analysis using the Enrichment Map plugin (Cytoscape© software) (Isserlin et al., 2014). Clusters of similar functional groups were then annotated using the AutoAnnotate plugin (Cytoscape© software) (Kucera et al., 2016). Default parameters were applied for each plugin.

### 2.4 Metabolomic analysis

The metabolomic analysis was performed on the same samples as the transcriptomic analysis. Extraction, derivatization, and injection were performed by the Plant Sciences Institute Metabolism‐Metabolome platform (Paris Saclay). Briefly, metabolites were extracted from 5 mg dry weight of sample dissolved in frozen Water:Acetonitrile:Isopropanol (2:3:3) containing Ribitol at 4 μg/mL as an internal standard. The solution was vortexed at 1500 rpm for 10 min at 4°C, then centrifuged at 13,500 rpm for 10 min. Aliquots (100 μL) of the supernatant were collected and 10 μL of myristic acid d27 at 30 μg/mL were added as an internal standard to lock the retention time. The extracts were dried in a Speed‐Vac for 4 h at 35°C and stored at −80°C. The same steps were then repeated with three blank tubes. Sample derivatization and analysis were performed by GC/MS as described in (Agilent‐Technologies, 2013; Fiehn, 2006; Fiehn et al., 2008): 1 μL of the sample was injected in splitless mode on an Agilent 7890B gas chromatograph coupled to an Agilent 5977A mass spectrometer. The column was a Rxi‐5SilMS from Restek (30 m with 10 m Integra‐Guard column, reference 13623‐127). Raw Agilent data files were analyzed using AMDIS (http://chemdata.nist.gov/mass‐spc/amdis/), and metabolites were identified using the Agilent Fiehn GC/MS Metabolomics RTL Library. Peak areas were determined using Masshunter Quantitative Analysis (Agilent) in splitless and split 30 modes, and integration was checked manually. Peak areas were normalized to Ribitol and dry weight. Metabolite contents are expressed in arbitrary units (semi‐quantitative determination). To calculate the fold change and the *p*‐value (*t*‐test), pairwise comparisons were performed after autoscaling (CIR–STR vs. CIR–IRR, IAC–STR vs. IAC–IRR, IAC–IRR vs. IAC–STR) using MetaboAnalyst (https://www.metaboanalyst.ca/). Differences in metabolite expression were considered significant at *p*‐value < .05. Joint pathway analysis with metabolomic and transcriptomic data were performed using MetaboAnalyst to identify the most significantly regulated pathways. Default parameters were used.

## 3 RESULTS AND DISCUSSION

Following stress, plant development is regulated at multiple levels, from genes to metabolites, which can ultimately modify growth and developmental patterns. Understanding the relationship between stress regulation and plant growth could help design new strategies to confer stress resistance, and identify high‐yielding plants (Bechtold & Field, 2018; Zhang et al., 2020). The development of drought‐tolerant rice varieties requires a better understanding of how the cellular and molecular mechanisms are regulated under stress (Ganie & Ahammed, 2021). The aim of the present study was to evaluate the response of two different genotypes to a moderate water deficit applied during the reproductive stage. The two genotypes were selected because, unlike other genotypes tested to date (Pereira de Lima et al., 2021), their showed different sensitivity to water stress that was maintained under contrasting experimental conditions. Their responses were therefore controlled by a genetic factor rather than an environmental factor. The two genotypes are good models to unravel the complexity of the biological mechanisms regulated under water deficit, and to explore the underlying characteristics that explain their respective adaptive versus non‐adaptive behavior.

**FIGURE 1:**
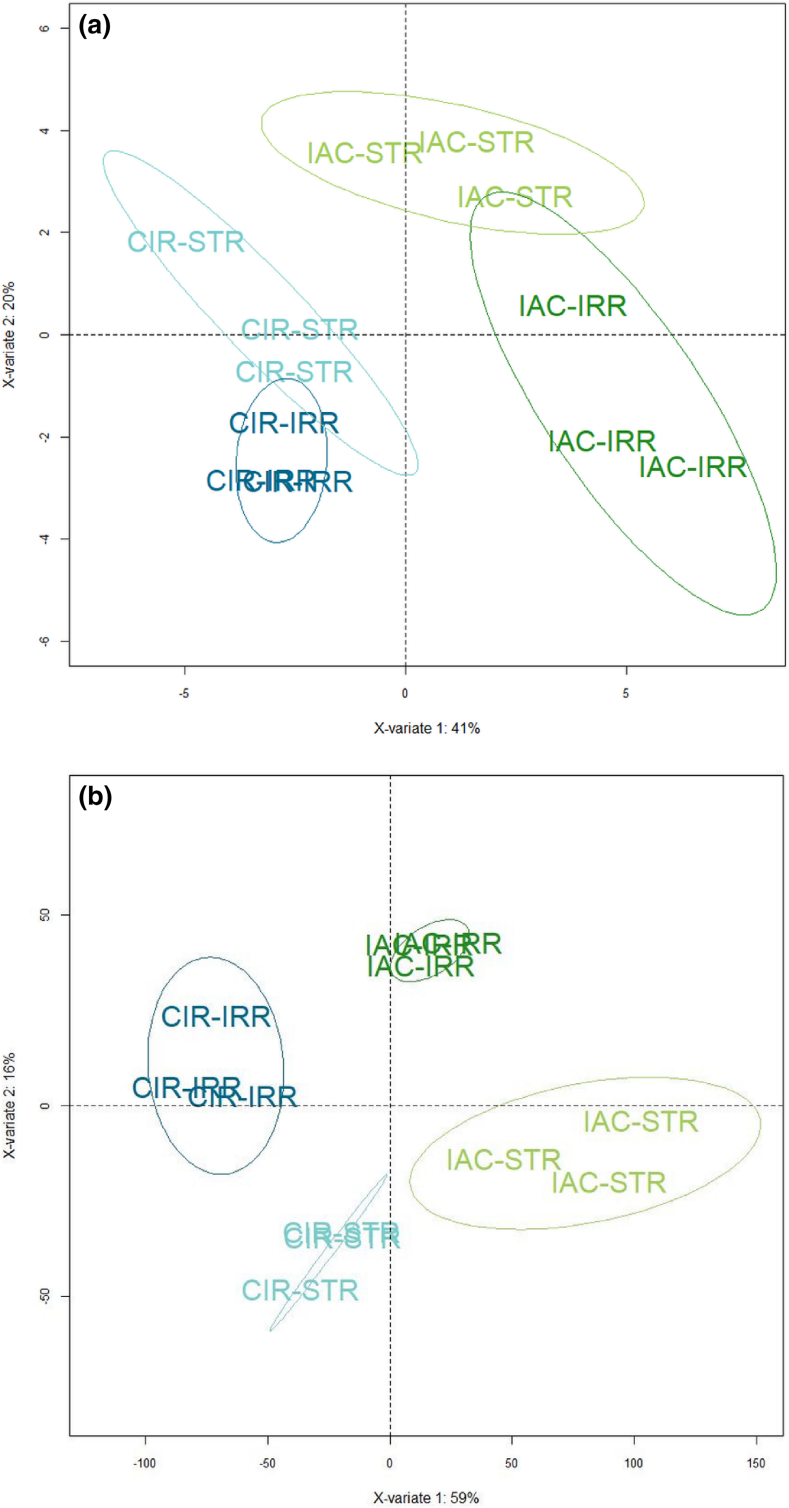
Partial least squares discriminant analysis of (a) phenotypic variables and (b) Multifactor genes in IAC and CIR in irrigated (IRR) and water‐stressed (STR) conditions.

Like the response of other plants to drought, the response of rice is complex involving changes at multiple physiological, biochemical, and molecular levels (Gupta et al., 2020; Melandri et al., 2020; Upadhyaya & Panda, 2019). In the present study, we conducted a multi‐level gene‐to‐phenotype analysis to investigate the response of the two selected genotypes according to the strategy described in Figure S1. The structure and the quality of the phenotypic (morphology, physiology, sugar content) and transcriptomic data were evaluated using the PLS–DA method. Like PCA, PLS–DA is a multivariate statistical method. However, it is a supervised method that uses sample classes. PLS–DA has been specifically recommended for use in omics data analysis (Ruiz‐Perez et al., 2020). The two PLS–DA plots, using the phenotypic (Figure 1a) and transcriptomic datasets (Figure 1b) showed that the two genotypes, CIR versus IAC, and the two water conditions, irrigation versus water deficit, were discriminated on the first two principal components PC1 and PC2. It shows that similar factors contribute to variations in both datasets. The variance of the data was explained without overlap by (i) the differences between the two genotypes (40% and 59% variability on PC1 for phenotypic and transcriptomic data, respectively), regardless of the water treatment, (ii) their respective response to the water treatment (20% and 16% variability on PC2 for phenotypic and transcriptomic data, respectively).

Phenotypic and metabolomic data were analyzed individually to identify the significant variables discriminating the two genotypes and/or the two water treatments. To distinguish between the molecular mechanisms regulated by each genotype, the transcriptomic data were analyzed using two strategies: (i) differential gene expression analysis to analyze the response of each genotype to water deficit versus irrigation, (ii) gene network analysis to highlight the specific biological processes regulated by each genotype under irrigation and water deficit. Correlation analysis between the gene networks and the phenotypic variables was then performed to identify relationships between gene regulation and developmental processes, and to identify hub genes. These results were then analyzed in a holistic way to identify the similar versus contrasted biological mechanisms regulated by each genotype, first under irrigation, and second, under water deficit. We then focused on certain key processes and genes that provide insights into the developmental process of each genotype.

### 3.1 IAC and CIR had contrasted growth profiles and gene regulation under irrigated conditions

**FIGURE 2:**
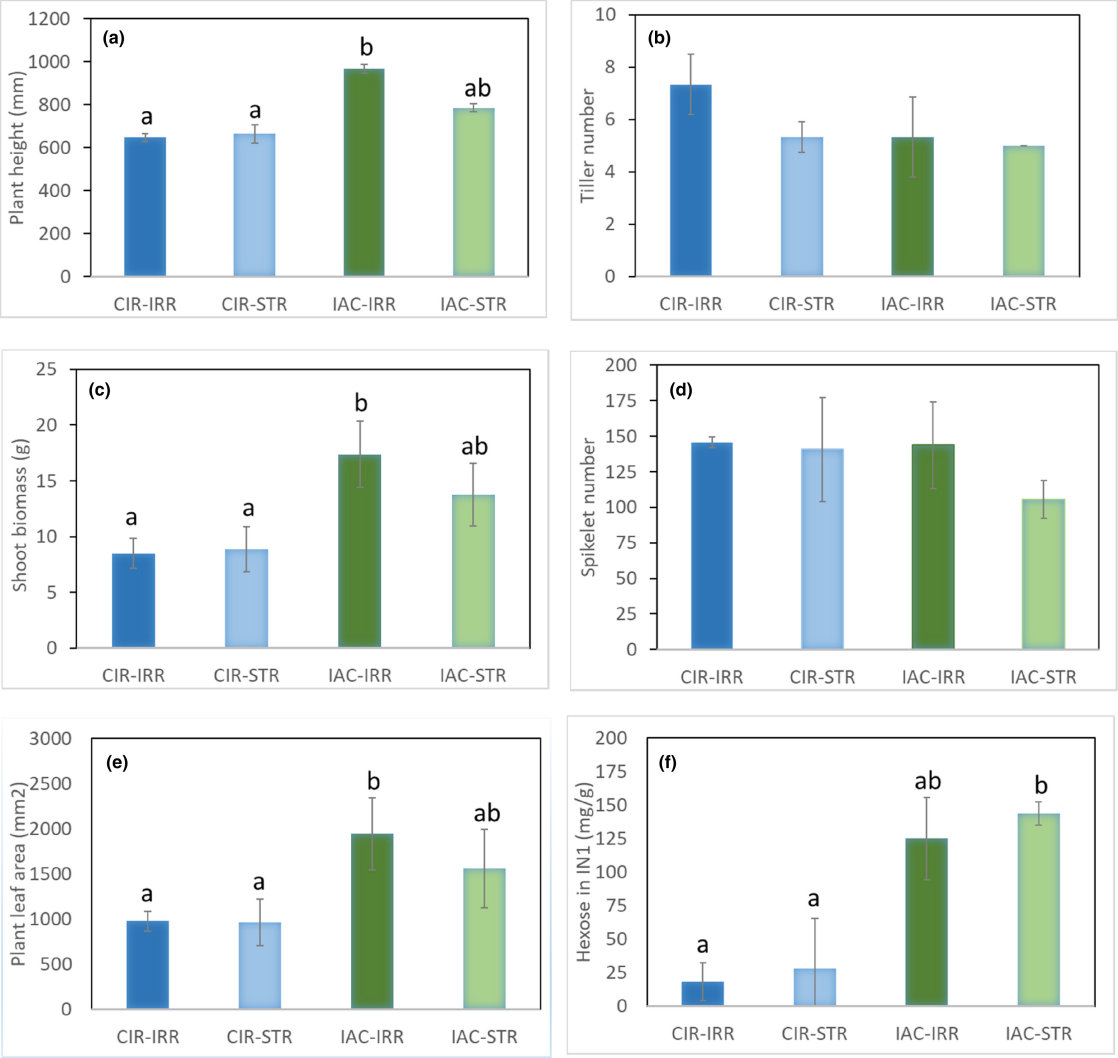
Phenotypic measurements of CIR (blue bars) and IAC plants (green bars) grown in irrigated (IRR) and water‐deficit (STR) conditions. (A) Plant height; (B) Number of tillers; (C) Shoot biomass; (D) Number of spikelets; (E) Plant leaf area; (F) Hexose in internode 1. Significance is indicated by a different letter for genotype × treatments.

A detailed analysis of the morphological and physiological variables identified the characteristics that explain the differences between the two irrigated genotypes (Table 1; Figure 2; Table S2). Plant height (97.6 vs. 194.3 cm), shoot biomass (8.5 vs. 17.4 g), and plant leaf area (0.98 vs. 1.94 m^2^) were all significantly lower in CIR–IRR than in IAC‐–IRR (Table 1; Figure 2A–C). The number of tillers per plant (Table 1; Figure 2D) did not differ between genotypes, unlike in the field (I. P. de Lima, personal communication), probably due to the low level of radiation in the growth chamber. At the level of the main tiller, total internode length and biomass differed significantly between the two genotypes, with lower values for CIR–IRR than for IAC–IRR (Table 1). However, no significant difference in the number of spikelets on the panicle was found between the genotypes (Table 1; Figure 2E), thereby highlighting a higher “reproductive/vegetative” ratio in CIR–IRR (Kato et al., 2011). These results confirmed those of Pereira et al (2021).

**TABLE 1:** Analysis of phenotypic variation between groups (genotype, treatment, genotype × treatment) using the Kruskall–Wallis test. For each group, mean values, adjusted *p*‐values (*p*‐adj < .05 are in bold) and effect size eta^2^[H] are shown. Effect size was considered small if 0.01 > eta^2^[H] < 0.06, moderate if 0.06 > eta^2^[H] < 0.14, large if eta^2^[H] ≥ 0.14.

Phenotypic variables		Genotype				Treatment				Genotype × treatment					
		CIR	IAC	*p*‐adj	eta^2^[H]	IRR	STR	*p‐*adj	eta^2^[H]	CIR–IRR	CIR–STR	IAC–IRR	IAC–STR	*p*‐adj	eta^2^[H]
Duration	Phyllo_Repr	6.68	8.70	**.0064**	0.6440	7.67	7.72	.7480	−.090	6.88	6.47	8.45	8.96	**.0470**	0.619
Organogenesis	Tiller_N°	6.33	5.17	.0861	0.1950	6.33	5.17	.1700	0.089	7.33	5.33	5.33	5.00	.1170	0.361
	TotBranch_N°	37.83	28.00	**.0250**	0.4030	35.67	30.17	.1500	0.108	39.33	36.33	32.00	24.00	.0656	0.526
	Spiklt_N°	143.17	124.67	.1090	0.1560	144.67	123.17	.1500	0.108	145.67	140.67	143.67	105.67	.1680	0.256
Morphogenesis	Shoot_Biom	8.68	15.55	**.0065**	0.641	12.93	11.30	.2310	−0.077	8.50	8.86	17.36	13.75	**.0405**	0.660
	TotLeaf_Area	968.54	1750.92	**.0065**	0.641	1459.23	1260.23	.6310	−0.077	975.53	961.54	1942.93	1558.91	**.0487**	0.609
	MainTil_Biom	2.17	4.03	**.0040**	0.731	3.37	2.83	.3370	−0.008	2.37	1.97	4.37	3.69	**.0261**	0.782
	Plant_Height	655.80	876.60	**.0040**	0.731	807.83	724.67	.5220	−0.059	648.00	663.70	967.70	785.70	**.0249**	0.795
	Pan_Biom	0.38	0.51	.2000	0.064	0.52	0.37	.0782	0.210	0.40	0.40	0.60	0.40	.0943	0.423
	Pan_Length	275.33	294.50	.2620	0.026	295.50	274.33	.2000	0.064	287.67	263.00	303.33	285.67	.3470	0.039
	FlagLeaf_Length	569.67	589.67	.5750	−0.069	628.17	531.17	.1280	0.132	615.33	524.00	641.00	538.33	.3840	0.006
	FlagLeaf_Width	15.83	20.17	**.0099**	0.566	18.00	18.00	1.0000	−0.100	15.33	16.33	20.67	19.67	.0754	0.486
	F‐1_Length	644.67	790.83	**.0374**	0.333	758.00	677.50	.2620	0.026	694.67	594.67	821.33	760.33	.1320	0.327
	F‐1_Width	14.17	18.33	**.0092**	0.578	17.00	15.50	.4640	−0.046	14.67	13.67	19.33	17.33	.0609	0.547
	IN1_Length	117.00	174.33	.2620	0.026	189.00	102.33	**.0040**	0.731	150.30	83.60	227.70	121.00	**.0216**	0.833
	IN1_Diam	4.48	5.30	**.0099**	0.566	5.18	4.60	.1070	0.160	4.70	4.30	5.70	4.90	**.0261**	0.782
	IN2_Length	88.33	179.67	**.0039**	0.734	144.17	123.83	.9360	−0.100	80.00	96.70	208.30	151.00	**.0169**	0.900
	IN2_Diam	4.73	6.00	**.0161**	0.479	5.62	5.12	.5210	−0.059	4.70	4.77	6.53	5.47	.0679	0.516
	IN3_Length	55.33	104.33	.0542	0.271	66.83	92.83	.2970	0.009	27.70	83.00	106.00	102.70	.1010	0.405
	IN3_Diam	4.83	6.00	**.0295**	0.374	5.70	5.13	.4680	−0.047	4.80	4.90	6.60	5.40	.0609	0.547
	INTot_Biom	0.19	0.30	**.0104**	0.556	0.27	0.23	.7490	−0.090	0.18	0.21	0.36	0.24	**.0476**	0.615
	INTot_Length	279.00	456.33	**.0038**	0.737	398.83	336.50	1.0000	−0.100	263.30	294.70	534.30	378.30	**.0151**	0.932
	Ped_Length	120.83	155.33	.7490	−0.090	197.67	78.50	.0547	0.269	127.67	114.00	267.67	43.00	.2180	0.179
	Ped_Diam	2.22	2.52	.5210	−0.059	2.70	2.04	**.0039**	0.734	2.43	2.00	2.97	2.07	**.0296**	0.748
	TotBranch_Length	135.86	123.86	.5220	−0.059	138.64	121.08	.1500	0.108	136.21	135.51	141.08	106.64	.3330	0.051
Biochemical composition	FL_Hex	6.46	11.92	.1090	0.156	7.38	11.00	.3370	−0.008	4.32	8.61	10.44	13.39	.2480	0.141
	FL_Sucr	58.35	53.27	.2620	0.026	52.63	58.99	.1090	0.156	55.09	61.61	50.17	56.37	.2820	0.103
	FL_Starch	1.76	0.39	**.0104**	0.556	0.79	1.35	.3370	−0.008	1.11	2.40	0.47	0.30	.0572	0.564
	IN1_Hex	23.16	134.38	**.0040**	0.731	71.73	85.81	.8730	−0.097	18.36	27.96	125.10	143.65	**.0378**	0.679
	IN1_Sucr	32.75	45.87	.2620	0.026	40.04	38.58	1.0000	−0.100	42.72	22.79	37.35	54.38	.1760	0.244
	IN1_Starch	2.63	2.48	.4230	−0.036	3.82	1.29	.2290	0.045	4.60	0.67	3.05	1.92	.4120	−0.016
	IN2_Hex	5.54	35.56	**.0163**	0.477	23.73	17.36	.3370	−0.008	3.67	7.41	43.80	27.32	**.0687**	0.513
	IN2_Sucr	48.00	41.83	.7490	−0.090	50.21	39.62	.5220	−0.059	50.40	45.61	50.01	33.64	.8630	−0.282
	IN2_Starch	40.57	6.38	.0782	0.210	22.00	24.96	1.0000	1.000	37.43	43.72	6.57	6.19	.3610	0.026
Physiologicalindicator	An	22.70	16.20	**.0250**	0.403	20.20	18.70	.5220	−0.059	23.70	21.70	16.70	15.70	.1410	0.308
	Tr	4.50	3.40	.0538	0.272	4.20	3.60	.2960	0.009	4.60	4.40	3.80	2.90	.1630	0.266
	Ci	289.90	278.00	.1280	0.132	290.00	277.90	.1500	0.108	290.00	289.80	290.00	266.00	1910	0.218
	An/Spad	0.48	0.33	**.0250**	0.403	0.42	0.39	.6310	−0.077	0.50	0.46	0.34	0.32	.1500	0.282
	InstWUE	5.00	4.90	.8730	−0.097	4.80	5.20	.1090	0.156	5.13	4.96	4.41	5.41	**.0273**	0.769
	CumWUE	3.70	3.80	.7490	−0.090	2.90	4.60	**.0040**	0.731	2.50	4.66	3.22	4.45	.0286	0.756
	SLA	262.90	251.80	.7490	−0.090	266.90	245.60	.0547	0.269	262.54	259.03	271.32	232.18	.1180	0.359

**FIGURE 3:**
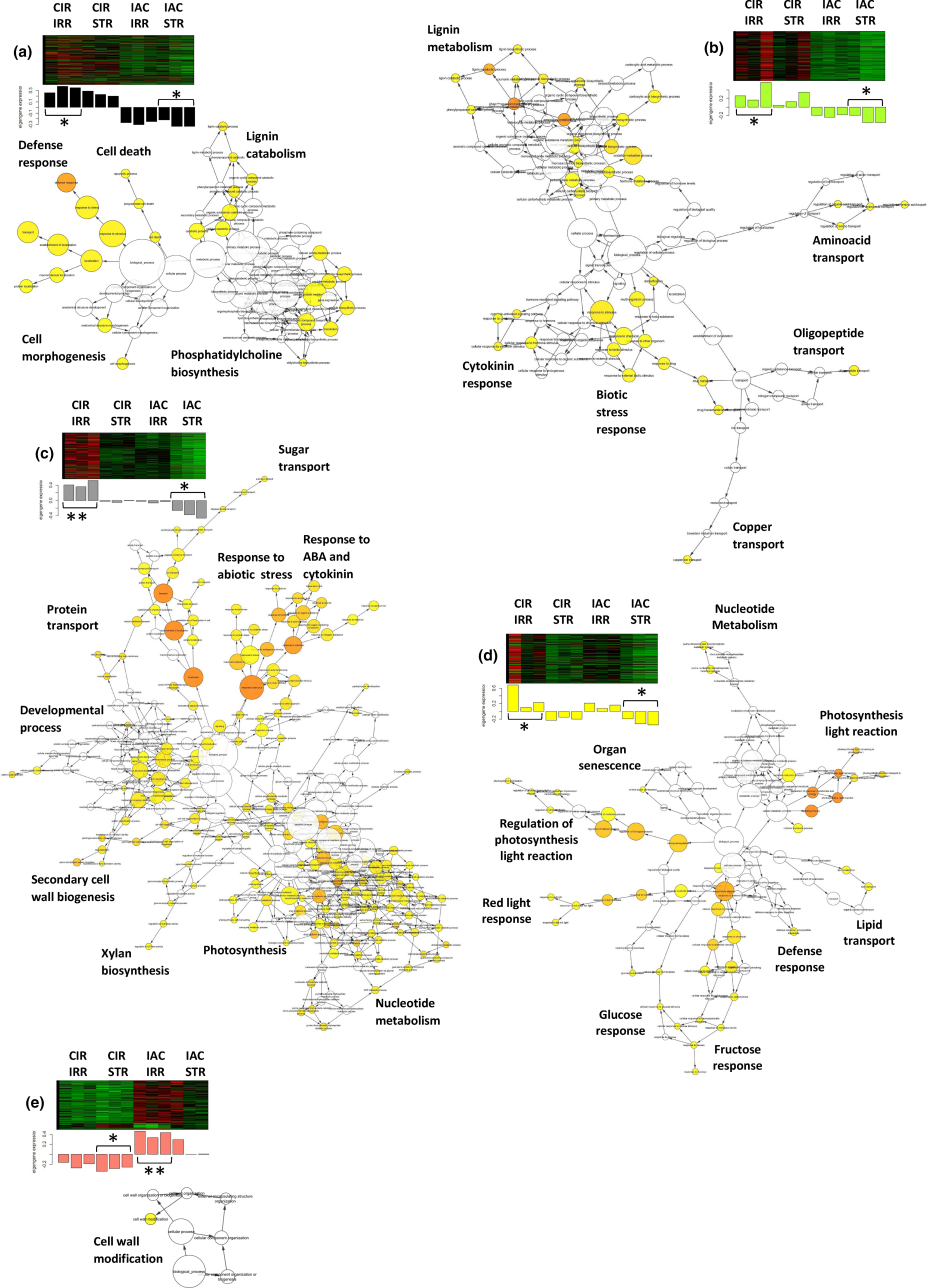
Representation of gene ontology enrichment of networks. Mean gene over‐regulation in CIR‐IRR: (a) black (*p*‐value = 2.1e‐2); (b) green‐yellow (*p*‐value = 1.6e‐2); (c) grey60 (*p*‐value = 3.4e‐4); (d) yellow (*p*‐value = 1.6e‐2); and IAC–IRR: (e) salmon (*p*‐value = 1.2e‐3). Histograms represent the mean gene expression profiles of each sample, and the significance of each genotype and water treatment (*p*‐value: ns >.05; * <.05; ** <.01; *** <.001). The color of the node represents the corrected *p*‐value. Colored nodes are significantly overrepresented, with more significant *p*‐values from yellow to orange. White nodes are not significantly overrepresented but they are the parents of overrepresented categories further down.

We used gene network analysis to identify the molecular mechanisms that were specifically regulated by each irrigated genotype (Figure 3). Under favorable water conditions, CIR over‐regulated more networks (Figure 3a–d) than IAC (Figure 3e), in contrast to its lower growth. The biological processes identified in the CIR networks were related to cell wall metabolism (cell morphogenesis, cell wall biogenesis, lignin metabolism), and photosynthesis. In contrast, a single process linked to cell wall modification was over‐regulated in IAC. It is not surprising that under favorable conditions, cell wall processes were regulated in both genotypes, since the cell wall structure is known to play a crucial role in plant growth, including in rice (Lin et al., 2016; Panda et al., 2021; Zhang et al., 2016). Cell wall regulation, and for CIR, photosynthesis, thus appeared to be the appropriate mechanisms to investigate. The molecular processes involved in these processes are discussed in Sections 3.2 and 3.3.

### 3.2 Under water deficit, only IAC showed reduced growth and higher levels of transcriptional activity and stress response compared to CIR

Drought affects rice physiology by modifying water use efficiency, relative water content, transpiration rate, stomatal conductance, net photosynthetic rate, internal CO_2_ concentration, photosystem II (PSII) activity, and the membrane stability index (Dash et al., 2018; Farooq et al., 2009; Mishra et al., 2018). In our experiment, the water deficit applied was moderate, consistent with most agricultural scenarios. Both genotypes showed increased cumulative water use efficiency (Table 1), evidence that they both suffered from the water deficit (Ullah et al., 2019). Rice has been shown to be sensitive to even a small decrease in available water (Nguyen et al., 2009). While no other physiological parameters were affected in either water‐deficient genotype, compared to irrigated conditions, significant variations were detected in some morphological variables. The expansion of the growing organs decreased systematically in IAC in water deficit conditions, whereas up and down variations were observed in CIR, i.e., in peduncle length, IN1 length, and IN2 length (Table 1).

**FIGURE 4:**
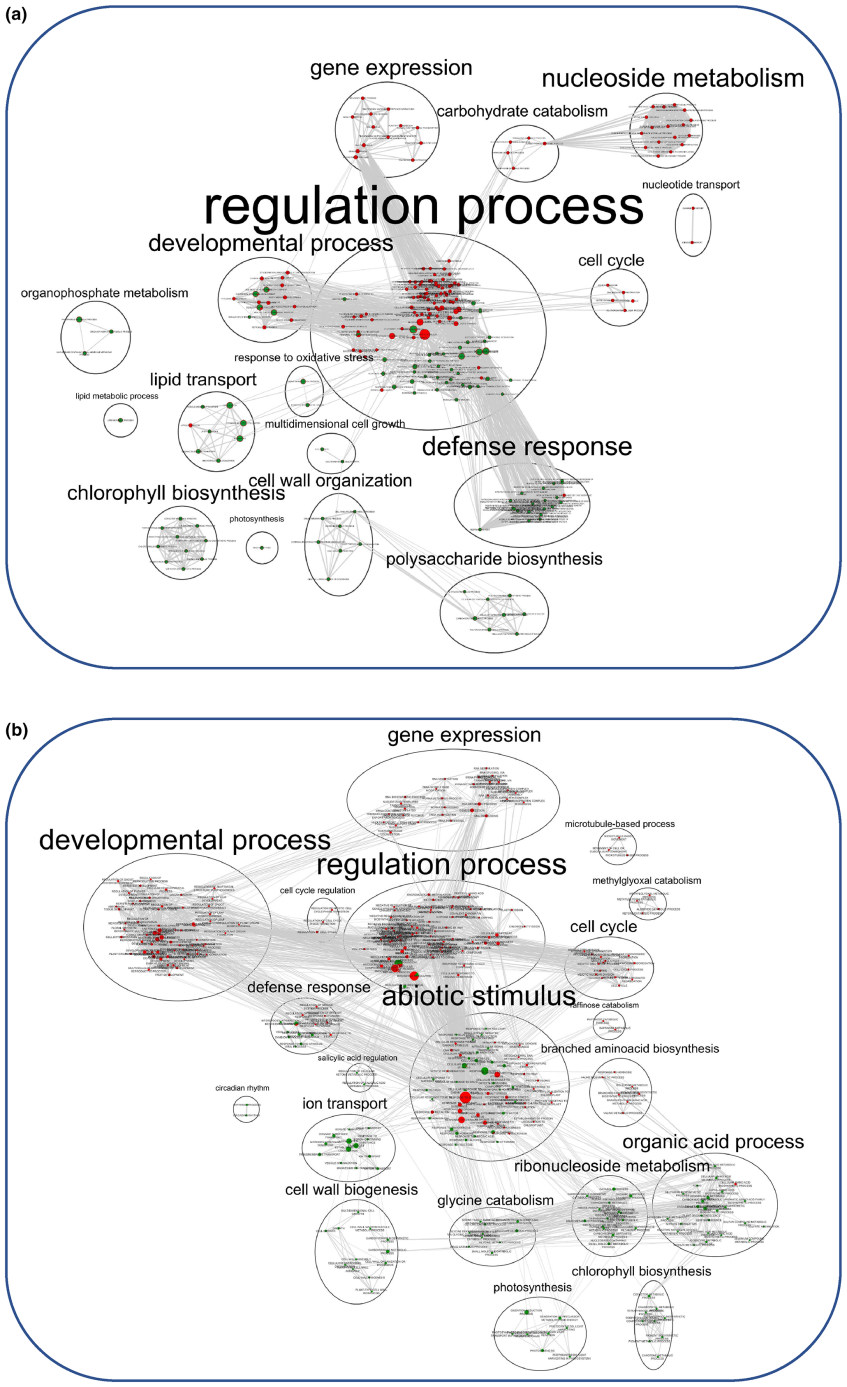
Enrichment map of biological processes up‐ and down‐regulated under water deficit in the CIR (a) and IAC (b) genotypes. The map shows the nodes representing GO enriched gene sets, connected by edges, representing similarity between the two gene sets. Nodes belonging to very similar biological processes are clustered, and labeled with a summarized name. A heatmap of up‐ and down‐expressed genes is shown for each cluster. Node color is proportional to the enrichment significance (*p*‐value < .05) of the corresponding up‐ (red) and down‐expressed (green) genes. Node size is proportional to the enrichment significance (*p*‐value < 0.05) of total up‐ and down‐expressed genes. Edge thickness is proportional to similarity between two nodes.

**FIGURE 5:**
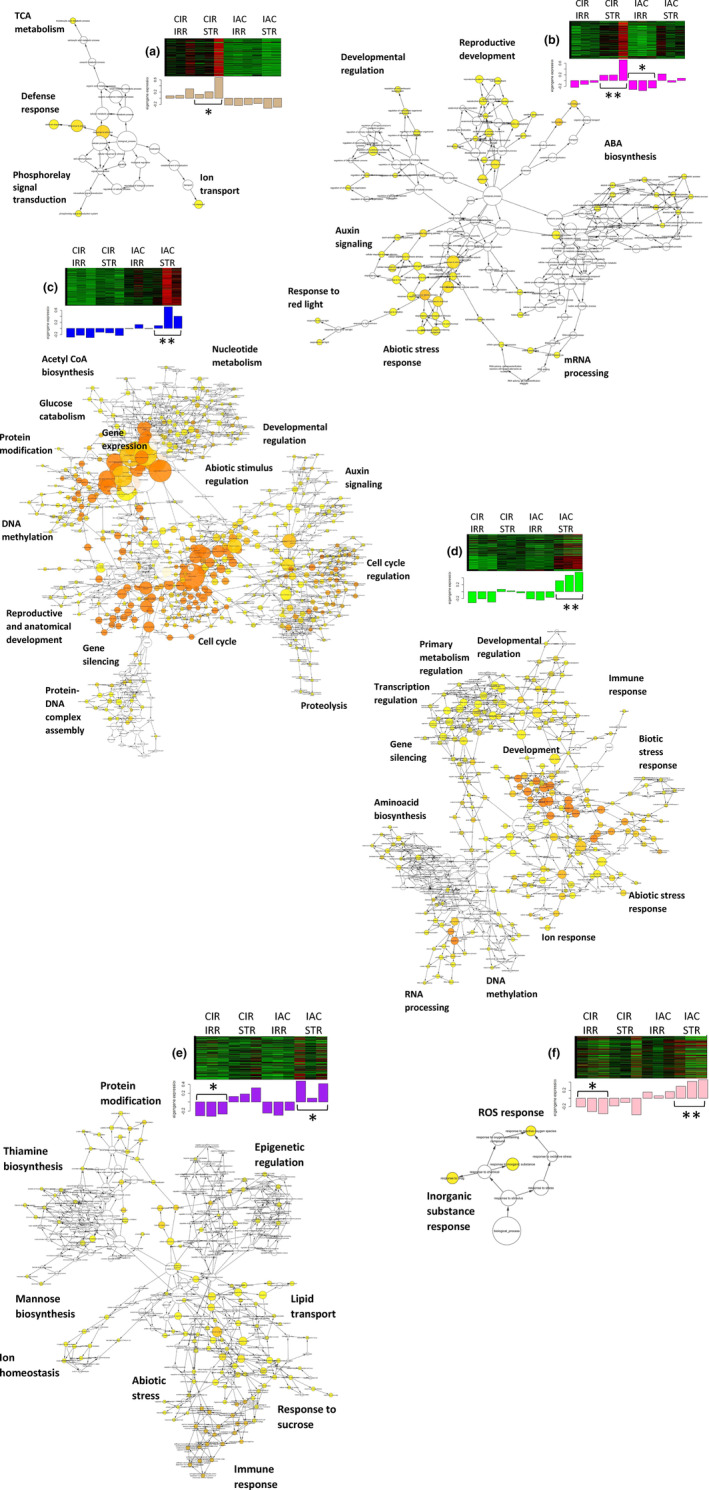
Gene ontology enrichment for gene networks over‐regulated under water‐deficit, for CIR–STR or IAC–STR. Histograms (a–e) represent the mean gene expression profiles of each sample (genotype and/or treatment). The red color represents the gene over‐expression and the green color represents the gene under‐expression. The stars represent the significance of the difference between groups (*p*‐value: ns >.05; * <.05; ** <.01; *** <.001). Map enrichment for each gene network representing CIR–STR (a) tan, (b) magenta; and IAC–STR (c) blue, (d) green, (e) purple, (f) pink. Colored nodes are significantly overrepresented, with more significant *p*‐values from yellow to orange. White nodes are not significantly overrepresented but they are the parents of overrepresented categories further down.

At the transcriptional level, the overall gene activity of IAC was higher than that of CIR, as indicated by the number of differentially expressed genes (1530 and 635 DEGs for IAC and CIR, respectively) (Figure 4; Table S2), and the number of specific gene networks (4 and 2, for IAC and CIR, respectively; Figure 5). Liang et al. (2021) also detected more DEGs in a susceptible genotype than in a moderately tolerant one. In CIR and IAC, differential gene expression analysis (water deficit vs. irrigation) revealed that they both regulated similar biological top gene ontology processes (Figure 4a,b): biotic and abiotic stress responses, gene expression and regulation, photosynthetic activity, primary and secondary metabolism, reproductive and developmental growth, and modification of the cell wall. These processes were similarly regulated in rice plants subjected to moderate to more drastic water deficits (Lenka et al., 2011; Liang et al., 2021; Plessis et al., 2015; Wilkins et al., 2016; Zhang et al., 2016). However, each genotype showed contrasting differences in the number of nodes (number of GO) and in the size of the nodes (number of genes involved in the process) of each cluster. Gene network analysis provided further insights into the specific molecular mechanisms regulated by each genotype (Figure 5).

In water‐deficit conditions, both genotypes up‐regulated genes involved in abiotic stress response (Figures 4 and 5). Regulation of this process has already been reported in rice under moderate water deficit (Liang et al., 2021; Plessis et al., 2015). Both genotypes over‐expressed high levels of dehydrin protein genes (log2fold 4 to 7; Table S2). These proteins belong to the late embryogenesis abundant (LEA) family, and are known to be drought resistant‐related genes. They are expressed in vegetative organs during periods of water deficit, in different tissues, and at different developmental stages in drought‐tolerant rice genotypes (Hanin et al., 2011; Verma et al., 2017; Wang, Pan, et al., 2011). Both genotypes expressed ABA‐related genes (IAC–STR, Table S2; CIR–STR, Figure 5b). In rice, like in other plants, the expression of dehydrin protein genes is positively regulated by ABA, and increasing ABA levels may benefit plants under stress conditions (Hanin et al., 2011; Ono et al., 1996; Verma et al., 2017; Xiong & Zhu, 2003). While both genotypes regulated abiotic stress processes, the transcriptional response was higher in IAC than in CIR (104 and 30 DEGs in IAC and CIR, respectively), including water stress (15 and 6 DEGs in IAC and CIR, respectively), and osmotic and oxidative stress (30 and 24 DEGs, respectively) only in IAC (Table S2). The processes identified in the two networks associated with IAC–STR confirmed its higher response to abiotic stress (Figure 5d,e), including water deprivation and osmotic stress (Table S2), and response to ROS (Figure 5f). ROS (radical oxygen species) production can result from over‐reduction of the electron transport chain (Melandri et al., 2020), or from an imbalance between ROS production and quenching, leading to oxidative stress, cell damage and ultimately, to plant death (Gill & Tuteja, 2010). IAC–STR also regulated more heat‐shock and chaperone proteins than CIR–STR (Table S2). These proteins protect other proteins from stress‐induced damage by regulating folding and unfolding affected by water deficit (Saibil, 2013). Therefore, the growth reduction in IAC under moderate water deficit was associated with higher transcriptional activity and stress response than in CIR. In addition, IAC regulated more networks than CIR (Figure 5), enriched in processes involved in the regulation of primary metabolism, cell cycle, developmental processes, transcriptional and post‐transcriptional regulation, and signaling. The molecular mechanisms involved in these processes are discussed below.

### 3.3 Regulation of cell wall metabolism and cell cycle processes

As described previously, transcriptional regulation of the cell wall was identified in the networks representing both CIR (Figure 3a–d) and IAC (Figure 3e). The importance of cell wall metabolism for plant growth is well established. It requires the production of cells with cell wall synthesis, proliferation to increase the number of cells, and expansion under the combined effect of turgor pressure and wall loosening (Hilty et al., 2021). In the present study, correlations were detected between the CIR networks and certain phenotypic variables related to developmental processes (Table 2). The four networks representing the irrigated CIR were largely dedicated to secondary cell wall biogenesis and lignin metabolism (Figure 3a–d). They were positively correlated with variables related to organogenesis‐related variables (higher total number of branches, spikelets and tillers), and negatively correlated with morphogenesis‐related variables (smaller organ dimensions). This suggests that in CIR–IRR, the transcriptional regulation of the cell wall is oriented toward organogenesis rather than morphogenesis. This hypothesis is supported by the much higher ratio of reproductive organs per unit of vegetative biomass in CIR–IRR (61.7) than in IAC–IRR (32.6), with as many branches and spikelets (145.7 vs. 143.7, in CIR–IRR and IAC–IRR, respectively), and a smaller main tiller (2.37 and 4.37 g, in CIR‐IRR and IAC–IRR, respectively). Conversely, the only IAC–IRR network, involved in cell wall regulation (Figure 3e), was positively correlated with 14 morphogenesis‐related variables (Table 2), with systematically higher values for the size of all its organ (Table 1). These results indicate that the growth process and cell wall regulation are closely related, and involve specific regulation by each genotype. Relationships between cell wall regulation and organogenesis/morphogenesis are not surprising, since the structure of the cell wall is the result of the regulation of its mechanical properties, which ultimately influence plant development (Hilty et al., 2021; Qiu et al., 2021).

**TABLE 2:** Correlations for network‐phenotype associations. The columns correspond to modules and the rows to phenotypic variables. Each cell contains the corresponding correlation values. Significant correlations (1 < R > −1; *p*‐values < .05) are colored: positive correlation: brown for 1.00 > Cor > 0.70; light brown for 0.69 < Cor > 0.60; negative correlation: green for −1.00 > Cor > −0.70; light green for −0.69 < Cor > − 0.60.

		IRRIGATION					WATER DEFICIENCY					
		CIR–IRR				IAC–IRR	CIR–STR		IAC–STR			
Function	Phenotypic variables	MEblack	MEgreenyellow	MEgrey60	MEyellow	MEsalmon	MEtan	MEmagenta	MEblue	MEgreen	MEpurple	MEpink
Duration	Phyllo_Repr	−0.81	−0.67	−0.56	−0.25	0.73	−0.71	−0.29	0.66	0.47	0.18	0.82
Organogenesis	Tiller_N°	0.52	0.43	0.63	0.59	−0.33	0.20	−0.27	−0.45	−0.46	−0.49	−0.44
	TotBranch_N°	0.69	0.76	0.76	0.49	−0.33	0.73	0.16	−0.81	−0.76	−0.40	−0.86
	Spiklt_N°	0.41	0.57	0.58	0.45	−0.05	0.58	0.12	−0.70	−0.69	−0.34	−0.66
Morphogenesis	Shoot_Biom	−0.85	−0.65	−0.51	0.02	0.89	−0.64	−0.36	0.42	0.15	0.06	0.70
	TotLEaf_Area	−0.82	−0.63	−0.48	0.02	0.85	−0.65	−0.40	0.42	0.15	0.03	0.69
	FlagLeaf_Length	−0.01	0.16	0.31	0.52	0.36	0.02	−0.24	−0.45	−0.45	−0.24	−0.11
	FlagLeaf_Width	−0.77	−0.63	−0.55	−0.11	0.78	−0.49	−0.07	0.38	0.23	0.25	0.59
	Pan_Biom	−0.45	−0.26	−0.01	0.33	0.74	−0.30	−0.43	−0.01	−0.37	−0.35	0.19
	Pan_Length	−0.29	−0.11	0.10	0.29	0.53	−0.23	−0.31	0.05	−0.23	−0.28	0.13
	F‐1_Length	−0.54	−0.31	−0.13	0.25	0.77	−0.45	−0.36	0.11	−0.05	−0.07	0.44
	F‐1_Width	−0.74	−0.59	−0.36	0.24	0.84	−0.69	−0.49	0.32	0.06	−0.04	0.65
	IN1_Length	−0.40	−0.19	0.17	0.46	0.66	−0.35	−0.55	0.05	−0.39	−0.54	0.11
	IN1_Diam	−0.69	−0.53	−0.19	0.30	0.84	−0.68	−0.68	0.29	−0.14	−0.34	0.51
	IN2_Length	−0.90	−0.80	−0.62	−0.12	0.91	−0.73	−0.37	0.56	0.23	0.06	0.77
	IN2_Diam	−0.71	−0.55	−0.36	0.03	0.90	−0.51	−0.31	0.22	−0.04	−0.02	0.50
	IN3_Length	−0.74	−0.71	−0.81	−0.52	0.52	−0.43	0.09	0.59	0.48	0.44	0.67
	IN3_Diam	−0.65	−0.50	−0.29	0.14	0.89	−0.46	−0.32	0.12	−0.13	−0.08	0.43
	INTot_Biom	−0.65	−0.57	−0.38	0.06	0.81	−0.53	−0.36	0.22	−0.01	−0.03	0.53
	INTot_Length	−0.87	−0.75	−0.53	−0.05	0.91	−0.67	−0.42	0.49	0.07	−0.08	0.64
	Ped_Length	−0.09	0.00	0.27	0.44	0.43	−0.14	−0.49	−0.27	−0.54	−0.50	−0.07
	Ped_Diam	−0.26	−0.03	0.28	0.53	0.61	−0.26	−0.64	−0.17	−0.57	−0.61	0.02
	TotBranch_Length	0.30	0.45	0.45	0.38	0.05	0.48	0.15	−0.62	−0.58	−0.21	−0.52
Biochemical composition	FL_Hex	−0.43	−0.49	−0.52	−0.41	0.38	−0.31	0.16	0.24	0.34	0.44	0.37
	FL_Sucr	0.38	0.14	−0.05	−0.37	−0.48	0.33	0.53	−0.19	0.17	0.41	−0.29
	FL_Starch	0.68	0.40	0.47	0.12	−0.56	0.35	0.01	−0.48	−0.35	−0.28	−0.60
	IN1_Hex	−0.71	−0.38	−0.37	0.03	0.67	−0.38	−0.15	0.44	0.19	0.11	0.54
	IN1_Sucr	−0.35	−0.06	−0.02	0.23	0.38	−0.17	−0.19	0.19	0.00	−0.08	0.25
	IN1_Starch	−0.14	0.02	0.28	0.59	0.23	−0.28	−0.58	0.04	−0.26	−0.49	0.11
	IN2_Hex	−0.83	−0.71	−0.68	−0.26	0.57	−0.58	−0.22	0.70	0.36	0.16	0.68
	IN2_Sucr	−0.08	0.18	0.07	0.34	0.10	0.18	0.03	−0.10	−0.22	−0.07	−0.09
	IN2_Starch	0.49	0.50	0.38	0.30	−0.54	0.51	0.11	−0.31	−0.34	−0.24	−0.51
Physiological indicator	CumWUE	−0.33	−0.37	−0.77	−0.74	−0.10	0.00	0.60	0.47	0.71	0.81	0.41
	Tr	0.60	0.58	0.59	0.21	−0.52	0.51	−0.06	−0.42	−0.56	−0.53	−0.72
	InstWUE	0.20	0.06	−0.13	−0.40	−0.52	0.06	0.37	0.27	0.61	0.44	0.11

The molecular mechanisms of the cell wall‐related processes regulated under irrigation were investigated in each genotype. In irrigated CIR, secondary cell wall metabolism was highly regulated in three out of its four networks (Figure 3a–c), thereby affecting the following processes: lignin, xylan, and cellulose metabolism, xylem and phloem pattern formation, cell wall biogenesis, and cell morphogenesis (Table S2). Lignins are only present in the secondary cell walls of specialized mature cells, which also contain celluloses, xyloglucans, and pectic polysaccharides (Cosgrove, 2018). After cellulose, lignins are the second main component of grass cell walls and account for 6%–12% of dry weight (Fry, 2011). Lignins play a key role in the secondary cell wall structure by enhancing rigidity, conferring resistance to pathogens and to mechanical stress, and enabling solute transport in the xylem (Brill et al., 1999; Chabannes et al., 2001; Jones, 2001). CIR–IRR expressed the CAD2 and CAD9 genes (Table S2), both of which belong to the last step of the phenylpropanoid pathway. They are involved in monolignol biosynthesis, and play major roles in lignin biosynthesis (Tobias & Chow, 2005). In rice internodes, high expression of OsCAD2 has been shown to be associated with high levels of lignin (Hirano et al., 2012), while the OsCAD2 rice mutant had reduced lignin content in its elongating stems (Zhang et al., 2016). In the present study, active regulation of lignin metabolism in CIR–IRR was supported by the over‐expression of 10 laccase proteins (Table S2). Laccase precursor proteins regulate lignin metabolism through lignin degradation and detoxification of lignin‐derived products, and are also involved in wound healing, maintenance of cell wall structure and integrity, and response to stress (Schuetz et al., 2014; Wang et al., 2015).

Cellulose is the main component of plant cell walls, and the most abundant polysaccharide produced by plants, and hence determines both cell shape and plant morphology (Zablackis et al., 1995). CIR–IRR had over‐activated cellulose‐related processes, including COBRA‐like proteins and glucuronosyltransferases, as well as several cellulose synthases (CesAs). CesAs genes are involved in cellulose and glucuronoxylan hemicellulose synthesis in secondary cell walls. Of the 6 over‐expressed CesAs in irrigated CIR, CesA6 and CesA9 are required for primary cell wall metabolism (Doblin et al., 2002), and CesA4, CesA7, and CesA8 are required for secondary cell wall metabolism (McFarlane et al., 2014). A joint‐pathway analysis using both metabolomic and transcriptomic data confirmed that the irrigated CIR over‐regulated the phenylpropanoid pathway leading to monolignol biosynthesis (false discovery rate of pathway enrichment = 6.2e‐5; data not shown), in contrast to IAC.

Secondary cell wall formation is regulated at many levels, including by transcription factors such as MYBs and NACs, and hormones (Didi et al., 2015). CIR–IRR regulated several putative MYBs (Table S2), as well as ABA and cytokinin response processes (Figure 3b,c). Both hormones are known to be involved in the regulation of secondary cell wall formation and/or other developmental processes such as cell cycle progression (Vanstraelen & Benková, 2012). Secondary cell walls are synthesized in the differentiated cell wall after cell growth has stopped, resulting in thicker walls with increased rigidity (Novakovic et al., 2018).

These results suggest that the irrigated CIR directed its cell wall metabolism toward strengthening tissue. Since no histological observations were available to support this hypothesis, stem density was roughly estimated for both genotypes. Based on the measured length and diameter of each internode (Table 1), the total volume of the stem was calculated by summing the volume of each internode considered as a perfect cylinder. The biomass of each internode was then summed to calculate the “stem biomass/stem volume” ratio (data not shown). The results confirmed that CIR–IRR had a higher stem density than IAC–IRR (36.0 and 19.6 mg/cm^3^, respectively). Furthermore, CIRAD 409 has been shown to allocate proportionally more assimilates to root system development under irrigated conditions, with a 68% higher root/shoot ratio than IAC 25 (Guimarães et al., 2020). Both factors could explain the slower shoot growth in CIR–IRR, i.e., because its smaller leaf area intercepts less light.

Under irrigated conditions, IAC regulated its cell wall in a similar way to CIR, but to a lesser extent and via a different mechanism (Figure 3e; Table S2). A transcriptional modification of the cell wall involving several pectinesterases was detected. Pectinesterases are proteins that catalyze the demethylesterification of homogalacturonans, and thus play an important role in regulating cell elongation in primary cell walls, leading to cell wall stiffening or loosening (Cosgrove, 2018; Micheli, 2001). Among the other genes identified, the two transcription factors SPL13 and LEUNIG have been shown to be involved in the regulation of flowering and cell growth. Similar to other squamosa promoter binding protein‐like, SPL13 is targeted by miR156 to regulate plant growth and development (Xie et al., 2006). In rice, SLP13 has been shown to regulate cell size by fine‐tuning microtubule and cell wall pathways, leading to improved development of secondary branches and more grains per panicle (Si et al., 2016; Yan et al., 2021). In Arabidopsis, the transcriptional corepressor LEUNIG has been shown to regulate cell expansion in the elongation zone (Geng et al., 2017), and cell wall modification for pectinaceous mucilage extrusion in seeds (Bui et al., 2011). Cell extensibility thus appears to be promoted in the irrigated IAC. This mechanism, which leads to an increase in cell size under the combined effects of turgor pressure and cell wall loosening (Hilty et al., 2021), is considered to be the main limiting factor for cell expansion (Baskin, 2005; Boudaoud, 2010; Geitmann & Ortega, 2009). These results underscore the fact that cell growth of the irrigated IAC was not completed at the time the samples were collected. These molecular mechanisms likely contribute to the greater growth rate of the IAC organs, compared to those of CIR.

In rice, cell wall plasticity plays a crucial role in plant development, especially when drought occurs during the reproductive stage. Cell wall plasticity is a trait that is considered to have high potential as a target for the development of high‐yielding varieties (Ganie & Ahammed, 2021). In our study, the cell wall process was down‐regulated in both water‐deficient genotypes (Figure 4), but by different mechanisms. Like in other plants, the properties of rice cell walls are known to be modified by drought, affecting flexibility and leading to growth inhibition (Ganie & Ahammed, 2021; Panda et al., 2021). Water stress causes low‐turgor pressure which, in turn, leads to a reduction or cessation of growth by reducing cell wall extensibility and cell expansion, two cellular processes that are indispensable for plant growth (Hsiao, 2000). These processes affect the flexibility of the expanding primary cell wall and/or the rigidity of the secondary cell wall (Kesten et al., 2017; Le Gall et al., 2015). Both cell wall expansion and rigidity are known to improve osmotic adjustment, and to prevent turgor loss and growth in many species, such as rice (Baldoni et al., 2016; Cal et al., 2013; Wang, Pan, et al., 2011). In our study, no over‐regulation of lignin‐related processes, which is an indicator of cell wall strengthening, were identified in either genotype as observed in many drought‐stressed plants (Le Gall et al., 2015; Lee et al., 2007; Morohashi & Russinova, 2019), including rice (Bang et al., 2019; Yang et al., 2006). However, both genotypes similarly down‐regulated processes related to microfibril organization, glucuronoxylan hemicellulose synthesis in secondary cell walls, loosening/extension of cell walls, and pectin and chitin metabolism (Table S2). Among these genes, many expansins, pectinesterases, glycosyltransferases, and COBRA genes were identified (Table S2). These proteins are involved in cell wall modification via deposition of cellulose and hemicelluloses in primary cell walls, and in cell wall plasticity, in a variety of plants (Braidwood et al., 2014; Le Gall et al., 2015; Liepman et al., 2010), as well as in rice (Jin et al., 2013; Wang, Pan, et al., 2011). Although cell wall plasticity is considered to be a critical trait for the rice plant to cope with drought (Ganie & Ahammed, 2021), it did not appear to be an adaptive mechanism in either genotype. However, since no deregulation of lignin metabolism was detected in CIR–STR, it is likely that the increased cell wall thickness, detected in the irrigated condition, was maintained under water deficit. Supporting this hypothesis, the “stem biomass/stem volume” ratio, calculated as described above, was still 1.5 times higher in water‐deficient CIR than in IAC (data not shown). This trait may give CIR an advantage in surviving water deficit conditions.

In contrast to CIR, the cell cycle process was up‐regulated in IAC–STR (Figure 5c), with the involvement of a microtubule‐based process composed of 18 over‐expressed kinesin‐like proteins (Table S2). These proteins play an important role in microtubule organization, organelle distribution, vesicle transport, and cellulose microfibril order. Their role in mitosis makes them essential for cell division and cell growth. Over‐regulation of kinesins could increase cell division, with microfibrils guiding cellulose synthase complexes for cellulose synthesis, or inhibit cell growth with microtubule reorientation in response to stress (Adamowski et al., 2019; Mirabet et al., 2018; Paredez et al., 2006). Under abiotic stress, a change in microtubule organization could control cell wall formation, ultimately affecting turgor pressure and cell growth (Ma & Liu, 2019; Wang, Zhang, & Chen, 2011). In the roots of rice exposed to drought, microtubule‐based movement was found to be up‐ or down‐regulated in two highly tolerant genotypes (Moumeni et al., 2015), and up‐regulated in a moderate tolerant one compared to in a susceptible one (Muthurajan et al., 2018). This cytoskeletal remodeling in IAC may contribute to the faster loss of turgor and the resulting reduction in its growth, described in the previous section. The reduction in growth is the result of cell division and/or cell wall properties, both of which are regulated under water deficit (Cosgrove, 2005; Granier et al., 2000).

### 3.4 Regulation of photosynthesis and metabolite biosynthesis

The two genotypes presented a similar SLA (Table 1), and therefore, a probably similar chlorophyll content per unit leaf area (Dingkuhn et al., 1998). However, the photosynthetic performance of the two genotypes differed. CIR had a higher assimilation rate (An) and ratio per unit of chlorophyll (An/Spad) (+40%) compared to IAC, regardless of the water conditions. Under irrigated conditions, CIR over‐regulated genes involved in photosynthetic activity and reaction to light (Figure 3c,d). Two important genes were identified among them, the ferredoxin‐NADP reductase (LNFR1) and the chloroplastic protein thylakoid rhodanese‐like (TROLL). LNFR1, an essential chloroplast enzyme involved in the final step of photosynthetic electron transfer, was shown to interact directly with TROLL (Yang et al., 2016). LNRF1 and TROLL interact to maintain high rates of photosynthesis, consistent with (Ort et al., 2015). They prevent over‐reduction of the entire electron transport chain (Juric et al., 2009), which leads to overproduction of reactive oxygen species. The higher regulation of the photosynthetic light reaction in irrigated CIR, compared with IAC, is consistent with the improved light‐harvesting performance of its flag leaf (An/Spad).

Higher photosynthetic activity in CIR did not result in higher sugar content in the internode 1, the pipe that transports assimilates from the flag leaf to the developing panicle. Conversely, IAC internode 1 contained six times more hexoses (Table 1) than CIR, regardless of the treatment applied. Hexoses are the sum of glucose and fructose, and both were shown to be over‐expressed in IAC–IRR compared to in CIR–IRR (Table 2). IAC internode 1 also contained 2.5 times more non‐structural carbohydrates (NSC), calculated as the sum of hexoses, sucrose and starch (data not shown). The local source/sink ratio, calculated according to (Fabre et al., 2020), was 0.48 and 0.69 in CIR–IRR and IAC–IRR, respectively. In other words, the sink strength can be considered as too weak in IAC, which means that the developing panicle is undersized compared to the size of the flag leaf, leading to the accumulation of NSCs in the internode. This is in line with the number of spikelets, which was identical in the two genotypes despite the fact the irrigated IAC had bigger tillers. In addition to the sugars targeted by biochemical analysis, metabolomic analysis showed that IAC–IRR synthesized other sugars at higher levels than CIR–IRR (Table 3), especially galactinol (+5.9 log2 fold change). Galactinol is a member of the raffinose family of oligosaccharides (RFOs), and is an alternative source of carbohydrate storage to starch (Sengupta et al., 2015). Overall, IAC–IRR differentially over‐expressed 10 out of the 13 metabolites (8 sugars and derivatives, 2 organic acids, 2 lipid compounds, 1 secondary metabolite) compared with CIR–IRR (Table 3). Higher metabolic activity may be required in irrigated IAC due to its higher growth rate, with, as a result, increased need for synthesis of structural or non‐structural compounds.

**TABLE 3:** Variations (log2Fold Change) and *p*‐values (*p*‐val) of significant metabolites in irrigated or water‐deficient CIR and IAC.

IAC–IRR/CIR–IRR			CIR–STR/CIR–IRR			IAC–STR/IAC–IRR		
Metabolites	Log2Fold change	*p*‐val	Metabolites	Log2Fold change	*p*‐val	Metabolites	Log2Fold change	*p*‐val
Galactinol	5.9	.017	D‐Malic acid	3.8	.044	Alpha‐Lactose	2.2	.002
Xylulose	3.9	.010	2‐Hydroxypyridine	0.8	.040	Beta‐Alanine	1.8	.004
Citric acid	3.7	.023				Azelaic acid	1.7	.005
Malic acid	3.5	.010				Ribose	1.5	.020
4‐Hydroxycinnamic	2.3	.030				2‐Hydroxypyridine	1.4	.023
Psicose	1.7	.000				Glyceric acid	1.2	002
Arachidic acid	1.7	.004				Lactulose	1.1	.023
Glucose	1.2	.026				Glycolic acid	1.1	.030
Mannose	0.9	.002				Myristic acid	0.92	.034
Fructose	0.5	.022				Vanillin	−0.76	.000
Trehalose	−1.5	.043						
Ethanolamine	−2.2	.040						
Lactitol	−2.3	.003						

Reduced photosynthesis under moderate drought has been reported in several studies (Barnaby et al., 2019; Plessis et al., 2015). However, under water deficit, no significant changes in the assimilation (An, An/Spad) and transpiration rates (Tr) were detected in the two genotypes, probably because they were measured early in the morning after the plant had recovered during the night. Conversely, the transcriptional activity involved in photosynthesis and reaction to light were down‐regulated in both genotypes compared to irrigated conditions (Figure 4), and down‐regulation was greater in IAC than in CIR (39 and 10 DEGs, respectively; Table S2). Among the genes regulated similarly in the two genotypes, a chlorophyll A–B binding protein (Os01G64960) was the most down‐expressed (log2Fold = −3.1 and 2.8 for IAC and CIR, respectively; Table S2). This gene was identified as one of the markers positively selected during the domestication of upland rice cultivars for adaption to water deficit (Zhang et al., 2016). As no decrease in photosynthetic activity was detected, the ROS over‐regulation in IAC (Figure 5f) was likely due to the imbalance between ROS production and quenching (Gill & Tuteja, 2010). ROS act as second messengers in sensing stress and can mediate rapid systemic signaling in response to stress (Choudhury et al., 2017). Although net CO_2_ assimilation was maintained in both water‐deficient genotypes when measured in the early morning, they both regulated photosynthetic processes at the transcriptomic level. Maintenance of photosynthesis has been linked to drought tolerance in rice (Panda et al., 2021). The transcriptional regulations observed in the two genotypes may thus indicate adaptive mechanisms.

In the previous section, we concluded that the reduced growth of water‐deficient IAC was likely due to the down‐regulation of its cell division and the modification of its cell wall properties. By reducing expansion prior to photosynthesis (Muller et al., 2011), a water deficit leads to the accumulation of structural and/or non‐structural sugars in rice and in many other crops (Cabuslay et al., 2002; Franck et al., 2006; Luquet et al., 2008; Rebolledo et al., 2012; Takahashi et al., 2020). Non‐structural compounds can be produced by primary metabolism (carbohydrates, amino acids, lipids), which sustains life processes and facilitates plant growth, and/or by secondary metabolism, which allows plants to respond and adapt to biotic and abiotic stresses. Primary metabolites can also be involved in stress response and act as compatible solutes (Agrawal, 2007; Ramakrishna & Ravishankar, 2011; Takahashi et al., 2020). In rice, accumulation of osmolytes such as water‐soluble carbohydrates, proline, soluble sugars, phenolics, and total free amino acids, increases under drought (Anjum et al., 2011; Keunen et al., 2013). Consistent with the higher stress response of the water‐deficient IAC, it over‐expressed more metabolites than CIR (10 and 2, respectively), than under irrigated conditions (Table 3). As expected, only a few were identified as secondary metabolites due to the use of the GC/MS technology, which mainly detects primary metabolites. Some of the metabolites over‐expressed in IAC–STR compared with IAC–IRR, have been shown to increase in response to abiotic stress, these include lactulose (Hu et al., 2016), glyceric acid (Kang et al., 2019) and β‐alanine (Kaplan et al., 2004; Parthasarathy et al., 2019; Rizhsky et al., 2004). Β‐Alanine was the only amino acid identified among the significant metabolites, while at the transcriptomic level, amino acid biosynthesis (22 DEGs, Table S2) and amino acid metabolism processes (Figure 5d) were over‐regulated in water‐deficient versus irrigated IAC. Amino acid accumulation has been measured in many water‐stressed plants (Batista‐Silva et al., 2019; Martinelli et al., 2007; Pérez‐Alfocea et al., 1993; Ranieri et al., 1989), but has been shown to be associated with high water stress in different rice genotypes (Barnaby et al., 2019). Of the two metabolites differentially expressed in water‐deficient versus irrigated CIR (Table 3), malic acid increased the most (3.8 log2 fold change). This organic acid, which belongs to the tricarboxylic acid (TCA) cycle, has been shown to be involved in drought tolerance in wheat and grass (Guo et al., 2018; Marcek et al., 2019). In contrast to results obtained in mild water‐stressed rice (Barnaby et al., 2019), sugar osmolytes did not increase in either genotype. Galactinol, glucose, and malic acid were found to be over‐expressed in IAC–IRR, compared to CIR–IRR, and none of them were detected when IAC–STR was compared to IAC–IRR (Table 3). This means that their level was maintained regardless of the water condition. Since glucose and malic acid are involved in osmotic balance (Chen et al., 2019; Saddhe et al., 2021), and galactinol protects cells from desiccation (Sengupta et al., 2015), these three compounds may provide some benefit to IAC–STR. Although the role of the compounds detected is still uncertain, these results show that the primary metabolism of IAC was more affected by water deprivation than that of CIR–STR. Some of its compounds may thus play a protective role at least under moderate water stress.

### 3.5 The development of the water‐deficient IAC was controlled by a gene regulatory network

Plants exposed to environmental changes need to fine‐tune their growth process and stress resistance by regulating complex transcriptional machinery (Braidwood et al., 2014). Both genotypes transcriptionally over‐regulated developmental processes (Figure 4) to a greater extent in IAC–STR, thereby participating in the development of reproductive (47 and 12 DEGs for IAC‐–STR and CIR–STR respectively; Table S2) and anatomical structures (84 and 61 DEGs in IAC–STR and CIR–STR, respectively; Table S2). In IAC–STR, this response was specifically represented in two networks (Figure 5c,d). The networks were enriched in genes involved in anatomical and reproductive development, in cell cycle and microtubule‐based processes, and in negative regulation of the cell cycle and nuclear division, as described above. More specifically, the corresponding genes were involved in transcriptional, post‐transcriptional and post‐translational regulations, RNA processing and splicing, gene silencing, DNA and histone methylation, and protein modification (Table S2). Plant responses are controlled by multiple transcription factors (TFs) that are linked to interacting genes and form complex gene regulatory networks (GRNs). GRNs include other regulatory factors such as microRNAs, hormones and/or chromatin‐modifying proteins. GRNs control responsive genes downstream, ultimately leading to phenotypic modifications such as changes in growth (Braidwood et al., 2014; Franciosini et al., 2017; Van den Broeck et al., 2020). The blue network (Figure 5c) was the only one composed of a large number of TFs (59) involved in development and/or stress response such as GRFs (Omidbakhshfard et al., 2015), MYBs and MYB‐related (Ambawat et al., 2013; Li et al., 2019), and ARFs (Li et al., 2016). The String database identified a significant level of physical protein–protein interactions in the genes of this network (*p‐*value < 1.0e‐16; Figure S3), thereby demonstrating the biological significance of the correlations identified between the genes of this network. Taken together, these results suggest that this network functions as a GRN. The blue network, as well as the green network (Figure 5d), were both negatively correlated with organogenesis‐related variables (Table 2), suggesting that the regulation of these genes is related to variations in the number of branches and spikelets on the IAC–STR panicle. In rice, a similar GRN was found to be correlated with crown root diameter (Kawakatsu et al., 2021).

Gene regulation involves not only molecular changes in regulatory sequences that can alter gene expression patterns (*cis*‐regulatory sequences), but also changes in mRNA, miRNA, and protein sequences (regulation in *trans*). Both types of modifications contribute differently to the generation of new phenotypes (Birchler & Veitia, 2010; Rieseberg & Blackman, 2010; Wittkopp et al., 2004). Several epigenetic mechanisms have been identified in the blue network including DNA methylation (C‐5‐cytosine specific DNA methylase, MET1/CMT3), core histone synthesis, post‐translational modifications (acetylation, methylation, phosphorylation), and RNA interference (Table S2). All of these mechanisms can alter gene function and ultimately the phenotype without modifying the DNA sequence (Berger, 2007; Bird, 2007; Grant‐Downton & Dickinson, 2005). DNA methylation is a stable, although reversible epigenetic mark that regulates gene expression during plant development and response to stress, and is heritable across generations (Iwasaki & Paszkowski, 2014). In the blue network, MET1 (CG context) and CMT3 (CHG context) were regulated, both of which are involved in DNA maintenance or de novo methylation (Law & Jacobsen, 2010). In Arabidopsis, regulation of these genes has been shown to strongly affect several phenotypic traits including plant height, number of branches and biomass (Bossdorf et al., 2010). This transgenerational epigenetic inheritance typically occurs in response to environmental cues and adaptive processes (Richards, 2008). While post‐transcriptional regulations were not analyzed in the present study, miRNAs that respond to water stress have already been identified in rice (Nadarajah & Kumar, 2019).

Hormonal signaling involved in development has been shown to be closely linked to GRN and miRNA regulation (Cimini et al., 2019; Li et al., 2020; Pajoro et al., 2014). The blue network includes auxin and gibberellin signaling, two hormones that are essential for growth regulation and cell elongation (Depuydt & Hardtke, 2011; Vanstraelen & Benková, 2012). Finally, both development and stress response are regulated by ROS signaling in interaction with epigenetic modifiers, such as DNA methylation levels and gene transcription (Berglund et al., 2017; Choi & Sano, 2007), hormones that control plant developmental processes, and stress responses (Chang et al., 2020; Gill & Tuteja, 2010; Tsukagoshi et al., 2010). The specific response to ROS regulation in IAC–STR was revealed by the pink network (Figure 5f). Like the blue and green networks, the pink network was negatively correlated with the total number of branches and spikelets, two organogenesis‐related variables, but positively correlated with seven morphogenesis‐related variables (Table 2). This supports our hypothesis of a central role for the blue network as GRN, as well as for the green and pink networks in the development of IAC–STR, through coordinated regulation of organogenesis and morphogenesis.

### 3.6 Core genes as potential central regulators of IAC and CIR growth

Hub genes, extracted using WGCNA, are referred to hereafter as core genes. Core genes are a small subset of genes that interact the most with other genes in a given network (Josephs et al., 2017; Mähler et al., 2017). It had been suggested that they are three times more likely to be essential than genes with fewer interactions. While high‐quality prediction requires identification of the other interacting genes, core genes are considered to be the best predictors of phenotypic traits because they summarize key information (Chateigner et al., 2019). In the present study, core genes were generated from networks highly correlated with eight phenotypic variables. Out of a total of 117 genes detected, the highest number (79 genes) was correlated with IN2 length, followed by the total number of branches (18 genes), IN2 diameter (15 genes), IN2 hexose (13 genes), and IN3 diameter (12 genes). Not surprisingly, no correlations were detected between the networks and the IN1 and peduncle dimension traits, as in the stress treatment, they were not systematically fully developed at the time of the dissection. Any short delay can lead to huge differences in their observed sizes. On the contrary, IN2, IN3, and branches were initiated during water deficiency, and were fully developed at the end of the experiment. It is likely that the gene regulation we detected was related to these organs. Only a few (5 genes) were related to phyllochron, shoot biomass (3 genes) and F‐1 width (1 gene). The functions of many of these genes remain unknown. Based on the mechanisms described above, among all the genes, 2 sets appear to be of great interest. Only genes with known functions are listed in Table 4, and are described below. Box plots of their gene expression levels can be seen in Figure S3.

**TABLE 4:** Selection of core genes.

High gene significance	Module membership	Gene ID	Name of gene	Function
IN2 length	Black	Os01g01570	Kinesin heavy chain	Microtubule motor activity
		Os01g68540	rho GDP‐dissociation inhibitor 1	Cell tip growth, root epidermal cell differentiation
		Os01g73750	Leucine Rich Repeat family protein, expressed	Protein ubiquitination
		Os02g33780	Serine‐aspartate repeat‐containing protein I precursor	NA
		Os03g51090	F‐box and DUF domain containing protein (OsFBDUF17)	Protein binding
		Os03g52230	Dynamin‐2B	GTPase activity
		Os03g54870	NLI interacting factor‐like phosphatase	NA
		Os03g63010	Plastid terminal oxidase	Oxidation–reduction process
		Os05g04220	Nitrogen regulatory protein P‐II (OsGLB)	Regulation of transcription, regulation of nitrogen utilization, regulation of fatty acid biosynthetic process
		Os05g04330	DNA methyltransferase protein	DNA methylation
		Os05g25400	RNA binding protein	rRNA processing
		Os06g01966	Auxin‐induced protein 5NG4, putative	Fatty acid biosynthetic process, response to cytokinin
		Os06g04690	OsFBX184—F‐box domain containing protein	Protein binding
		Os06g11300	CRR4	Protein binding
		Os06g20020	ZOS6‐03—C2H2 zinc finger protein	Nucleic acid binding
		Os06g49840	OsMADS16—MADS‐box family gene with MIKCc type‐box	Regulation of transcription, cell differentiation
		Os06g50400	Expansin precursor	Plant‐type cell wall organization
		Os07g06500	OsFBL34—F‐box domain and LRR containing protein	Protein binding
		Os08g29530	Double‐stranded RNA binding motif containing protein	Pre‐miRNA processing
		Os08g29590	Zinc finger, C3HC4 type family protein	Zinc ion binding
		Os10g2318	Cytochrome P450	Oxidation–reduction process
		Os10g36620	Hydroxyproline‐rich glycoprotein DZ‐HRGP	NA
		Os11g38500	OsFBDUF62—F‐box and DUF domain containing protein	Protein binding
		Os11g42040	Non‐TIR‐NBS‐LRR type resistance protein, putative	ADP binding
Total branch number	Blue	Os01g36600	PPR repeat domain containing protein	DNA repair, cell‐cycle regulation
		Os02g37430	LSM domain containing protein	Spliceosomal complex assembly
		Os03g58330	BHLH transcription factor (AtUNE12)	Regulation of transcription, regulation of defense response
		Os04g56970	Tubulin/FtsZ domain containing protein	Microtubule‐based movement
		Os06g50910	Phosphatidylinositol kinase and FAT containing domain protein (AtATR)	Cell cycle, regulation of DNA repair, multicellular organism reproduction
		Os09g28200	Heat stress transcription factor B‐4c (HSFB4C)	Regulation of transcription, regulation of HSPs

	Pink	Os02g32530	SAM domain family protein	Protein channel activity
		Os03g05390	Citrate transporter protein	Transmembrane transport
		Os07g44310	Heat shock protein DnaJ	NA
		Os04g37920	FAD binding domain of DNA photolyase domain containing protein (AtCRY1)	Blue light signaling pathway, regulation of unidimensional cell growth
		Os10g39670	Protein kinase family protein (AtBSK1‐2)	Protein phosphorylation, brassinosteroid mediated signaling pathway
		Os04g52000	Protein phosphatase 2C	protein dephosphorylation
		Os08g25380	Serine/threonine‐protein kinase BRI1‐like 1 precursor (OsBRL3)	Protein phosphorylation, brassinosteroid mediated signaling pathway
		Os01g67490	OTU‐like cysteine protease family protein	NA
		Os11g06170	bZIP transcriptional activator RSG (AtVIP1)	Regulation of transcription, negative regulation of cell differentiation
Os01g43844		Cytochrome P450 72A1	Oxidation–reduction process

The first gene set belongs to the black network (Figure 3a), and represents cell wall metabolism in CIR–IRR, confirming the importance of this process. These genes were significantly correlated with the length of IN2, i.e., the most contrasted phenotypic variable between CIR–IRR and IAC–IRR. IN2 is associated with the second leaf (i.e., the first leaf below the flag leaf), and its entire development and elongation stages took place during the water deficit period. Most of the 59 core genes identified in the black network have unknown or poorly defined functions, and 32 were not annotated at all. Most striking was the presence of 21 F‐box proteins. These proteins are involved in the regulation of different plant development processes, including photomorphogenesis, circadian clock regulation, self‐incompatibility, floral meristem, and floral organ identity determination (Moon et al., 2004; Smalle & Vierstra, 2004; Sullivan et al., 2003). In rice, more than 900 F‐box proteins have been identified and classified into 10 families (Jain et al., 2007), but functions have only been attributed to a few F‐box proteins. In Arabidopsis, 3 F‐box proteins have been shown to physically interact with four PAL isozymes, key proteins of the phenylpropanoid pathway, and to mediate their proteolytic turnover via the ubiquitination‐26S proteasome pathway (Zhang et al., 2013). Two transcription factors, the nitrogen regulatory protein P‐II (OsGLB) and the MIKCc type‐box (OsMADS16), were detected among the other core genes. OsGLB is involved in carbon and nitrogen sensing, and regulates the key enzyme N‐acetyl glutamate kinase (NAGK) of the arginine biosynthetic pathway (Ferrario‐Méry et al., 2006). Little is known about its function. OsMADS16 has been identified as being involved in cell differentiation, particularly in floral organ identity and meristem fate in rice (Ohmori et al., 2009). Compared with IAC, F‐box proteins were over‐regulated and the two TFs were down‐regulated in CIR, regardless of the water regime concerned. Although the limited amount of information prevents us from concluding on the role of these genes, they are likely to be important actors in cell wall regulation and cell growth in the development of irrigated CIR.

The second set of genes belongs to two networks, the blue network (Figure 5c) identified as a GRN involved in IAC–STR developmental process, and the pink network (Figure 5f) involved in its response to ROS. Among the core genes, three transcription factors have been shown to be involved in developmental processes and/or stress response. In Arabidopsis, increased turgor pressure was shown to lead to higher expression of the bZIP transcriptional activator RSG (AtVIP1), which decreased after the cell had adapted to changes in turgor pressure (Tsugama et al., 2012). The heat stress transcription factor B‐4c (HSFB4C) and the basic helix–loop–helix bHLH059 (AtUNE12) are both involved in the defense response, but their functions remain to be elucidated (Guo et al., 2016; Heim et al., 2003). Two core genes that belong to the brassinosteroid receptor family, the serine/threonine protein kinase brassinosteroid‐insensitive 1 (BRI1)‐like 1 precursor (BRL3), and the brassinosteroid (BR)‐signaling kinase 1‐2 (BSK1‐2). Brassinosteroids (BRs) are growth‐promoting hormones that interact with auxin to regulate a wide range of physiological and developmental processes including cell elongation, seed germination, stomatal formation, vascular differentiation, plant architecture, flowering, stress resistance, male fertility, and senescence (Nemhauser et al., 2004). BRs depend to a greater extent on transcription factors to regulate developmental processes (Li, 2010), and their receptors are involved in fine‐tuning the direction and rate of cell division (Tong & Chu, 2018). The homeostasis of BRs is critical because the control of plant development is regulated in a dose‐dependent manner, with limited application of BRs resulting in increased in growth, whereas higher exposure is detrimental (Clouse et al., 1996). Interestingly, the expression of BRL3 and BSK1‐2 was significantly decreased in IAC‐–STR, compared to IAC–IRR, and remained high in CIR–IRR and CIR–STR. BR signaling is likely to play a critical role in regulating the cell growth in IAC–STR.

The core genes identified in the networks provide further insights into the mechanisms controlling the growth of irrigated CIR, on the one hand, and water‐deficient IAC, on the other hand. More in‐depth analysis using tissue imaging and reverse genetics is now required to understand their precise role in regulating the plant phenotype.

## 4 CONCLUSION

**FIGURE 6:**
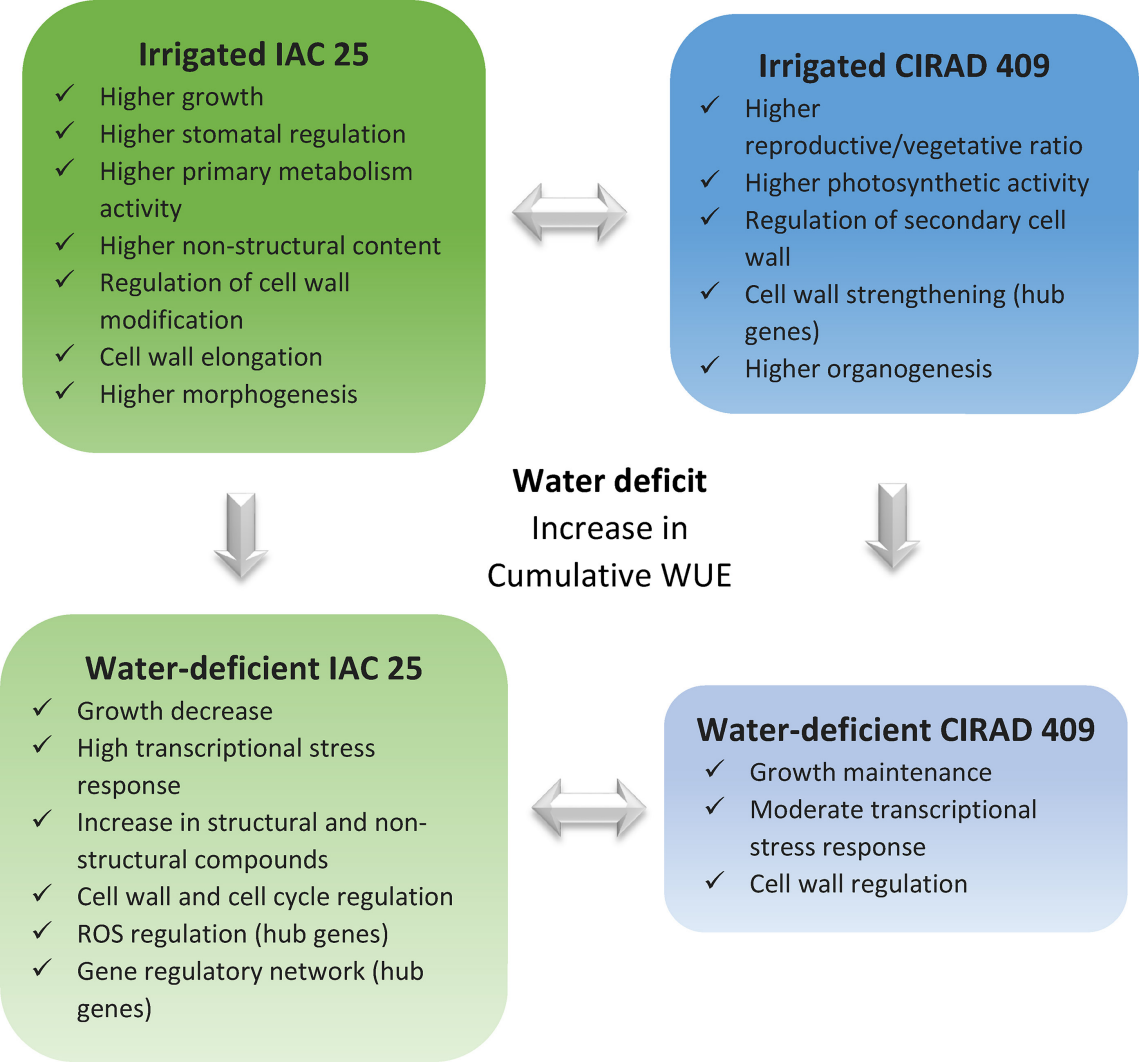
Multi‐level response of CIRAD 409 and IAC 25 to moderate water deficit during the reproductive stage.

We performed a prospective and holistic analysis of the biological mechanisms that regulate the growth of two contrasting rice genotypes during the reproductive stage, under limiting and non‐limiting water conditions. To unravel the complexity of the mechanisms driving the developmental processes of each genotype under moderate water stress conditions, we collected and analyzed data concerning different levels of plant organization, from gene expression to the whole plant (Figure 6). Compared to IAC 25, CIRAD 409 has constitutively: 1—a better photosynthetic performance; 2—a denser shoot structure and a higher root/shoot ratio (Guimarães et al., 2020) associated with slower shoot growth; 3—a higher organogenetic and a lower morphogenetic capacity (ability to generate new organs vs to expand existing ones), resulting in a shorter plant with a high number of spikelets per tiller. Significant core genes involved in cell wall metabolism were identified in CIRAD 409 in non‐limiting conditions, i.e., under irrigation, highlighting the fact that its internode length was at least partially regulated at the transcriptional level. Conversely, the irrigated IAC 25 had higher metabolic activity, a higher morphogenetic capacity with bigger organs, and a more rapid shoot growth, which could be boosted by cell elongation.

Under moderate water deficit, CIRAD 409 was able to maintain its overall shoot growth and panicle development whereas in IAC 25, the same traits were reduced or blocked. In IAC 25, we hypothesize that its photoassimilates were poorly used in the sink organs, leading to significant accumulation of non‐structural compounds in the stem internodes in parallel with changes in metabolic activity. In addition, the regulation of transcriptional activity and the cell cycle were deeply modified under water deficit conditions. More strikingly, the identification of a GRN, and of core genes, suggests that the growth of the water‐stressed IAC 25 was controlled by the regulation of a complex transcriptional, post‐transcriptional, post‐translational and hormone network. Finally, it is important to note that the growth maintenance strategy displayed by CIRAD 409, driven by sink strength, is certainly adapted in the case of a moderate and time‐limited water deficit, but could be risky under more severe water stress. Given the diversity of drought conditions, there is no single biological adaptive response.

Although the present study involved only two genotypes, it revealed fundamental processes that drive rice development and its response to water deficit: in particular, the biosynthetic and regulatory pathways of cell wall biosynthesis, in both favorable and limiting water conditions. These key features determine the mechanical properties of the cell wall and thus plant development, organ expansion and turgor maintenance under water deficit. Furthermore, our study questions the genericity of the antagonism between morphogenesis and organogenesis as observed in the two genotypes.

Finally, our results highlight the importance of using multi‐level analysis to tackle the complex mechanisms involved in the regulation of plant development in response to stress. Gene network analysis is a powerful tool to reveal the underlying molecular mechanisms and potential biological markers. Of course, such a study has its limits. Our current knowledge of plant regulatory processes remains partial and fragmented, and our interpretations are intended as hypotheses to be explored rather than as conclusions. However, this study has helped us to deepen our understanding of the relationships between the developmental processes involved in plant growth and the regulatory mechanisms of the stress, and paves the way for new strategies to confer stress resistance and to identify high‐yielding plants.

## CONFLICT OF INTEREST STATEMENT

All authors declare that they have no conflicts of interest.

## Supporting information


Figure S1

Figure S2

Figure S3

Table S1

Table S2

## Data Availability

Fastq files of the sequenced libraries were deposed in the in publicly accessible NCBI's Sequence Read Archive (SRA) under the accession number: SAMN27520494, SAMN27520495.
