# Supplementary material for: A multi‐level approach reveals key physiological and molecular traits in the response of two rice genotypes subjected to water deficit at the reproductive stage
Source: Plant Environ Interact. 2023 Sep 15;4(5):229–57. doi: 10.1002/pei3.10121 (PMC10564380; doi:10.1002/pei3.10121)
Supplement: Supplementary file 1 — Figure S1 [file PEI3-4-229-s005.docx]

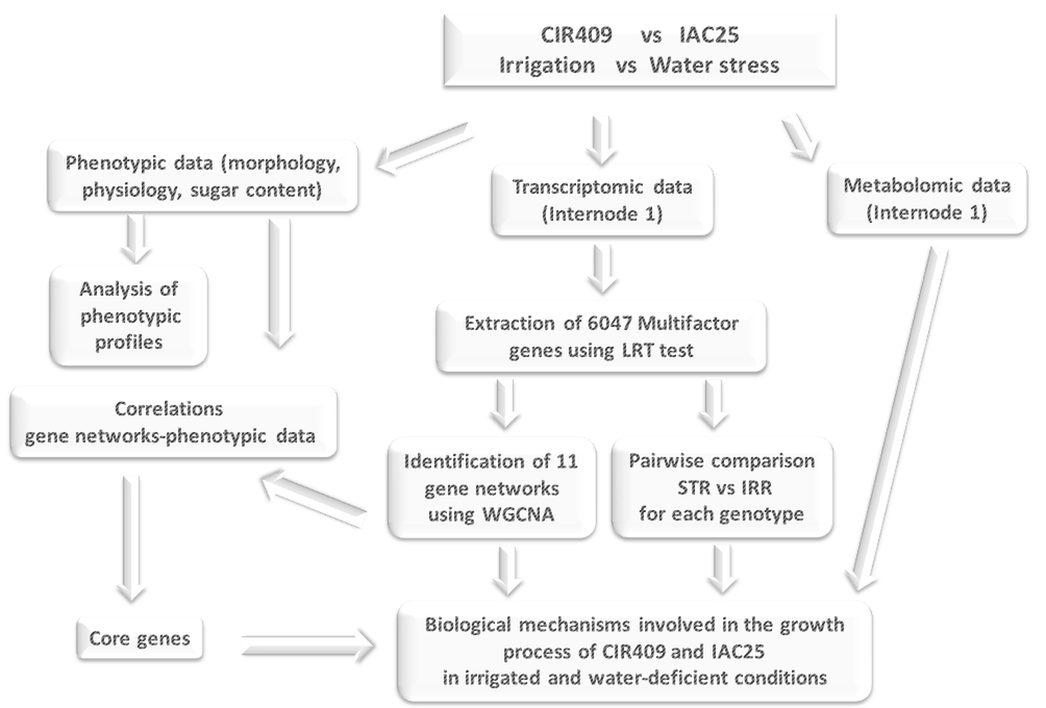


**Figure S1**: Strategy applied to study the multi-level response of 2 rice genotypes (CIR 409, IAC25) under a moderate water deficiency applied during the reproductive phase
