# Supplementary figures and images for: A multi‐level approach reveals key physiological and molecular traits in the response of two rice genotypes subjected to water deficit at the reproductive stage
Source: Plant Environ Interact. 2023 Sep 15;4(5):229–57. doi: 10.1002/pei3.10121 (PMC10564380; doi:10.1002/pei3.10121)

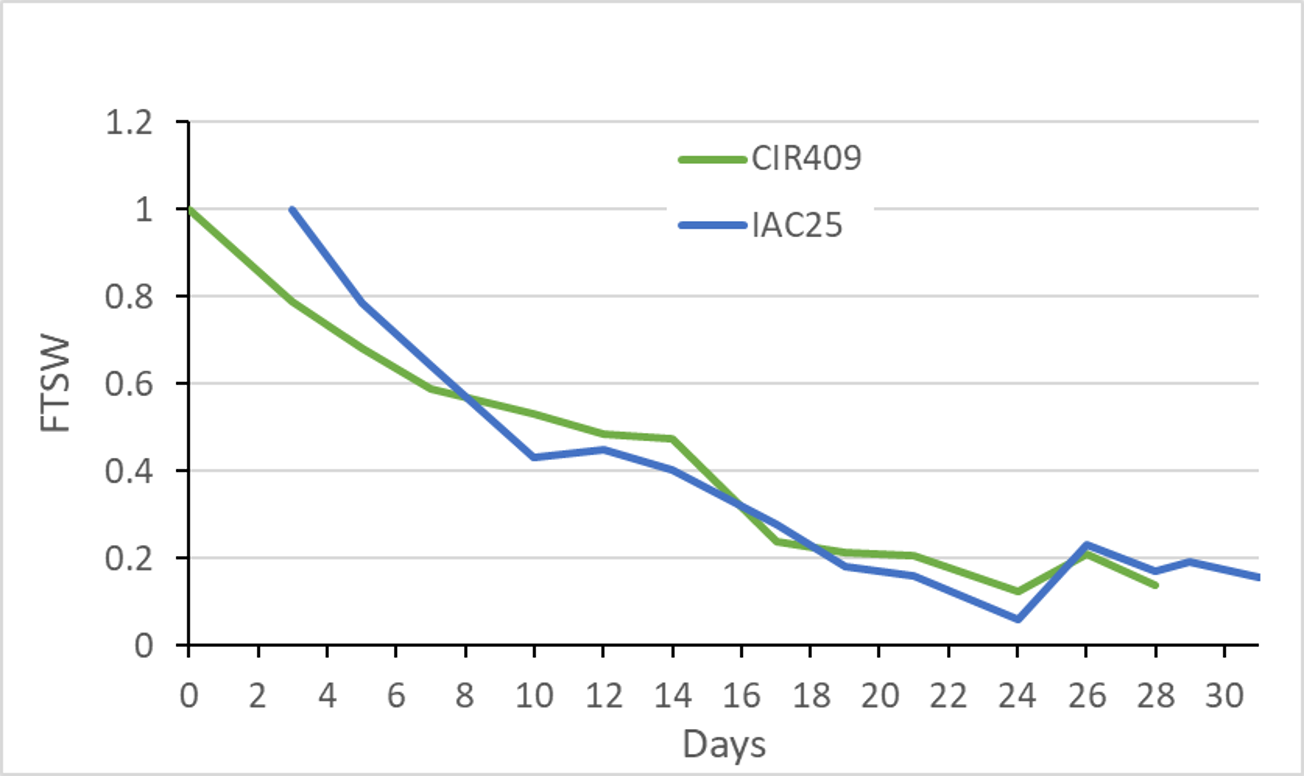


Figure S2 : Time evolution of the FTSW during the water deficit application for each genotype

Supplement: Supplementary file 2 — Figure S2 [file PEI3-4-229-s003.docx]

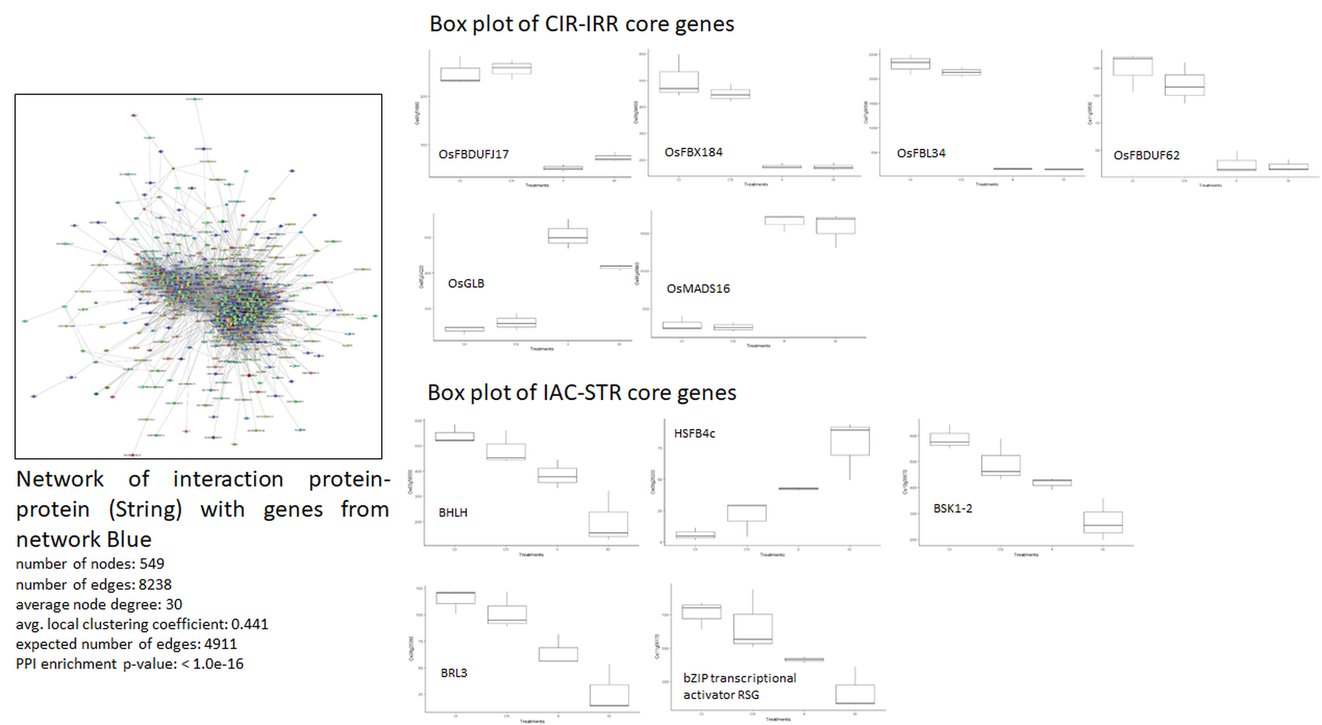

Supplement: Supplementary file 3 — Figure S3 [file PEI3-4-229-s001.zip › S5 Figure.docx]
