# Supplementary material for: A multi‐level approach reveals key physiological and molecular traits in the response of two rice genotypes subjected to water deficit at the reproductive stage
Source: Plant Environ Interact. 2023 Sep 15;4(5):229–57. doi: 10.1002/pei3.10121 (PMC10564380; doi:10.1002/pei3.10121)
Supplement: Supplementary file 3 — Figure S3 [file PEI3-4-229-s001.zip › S5 Figure_caption.docx]

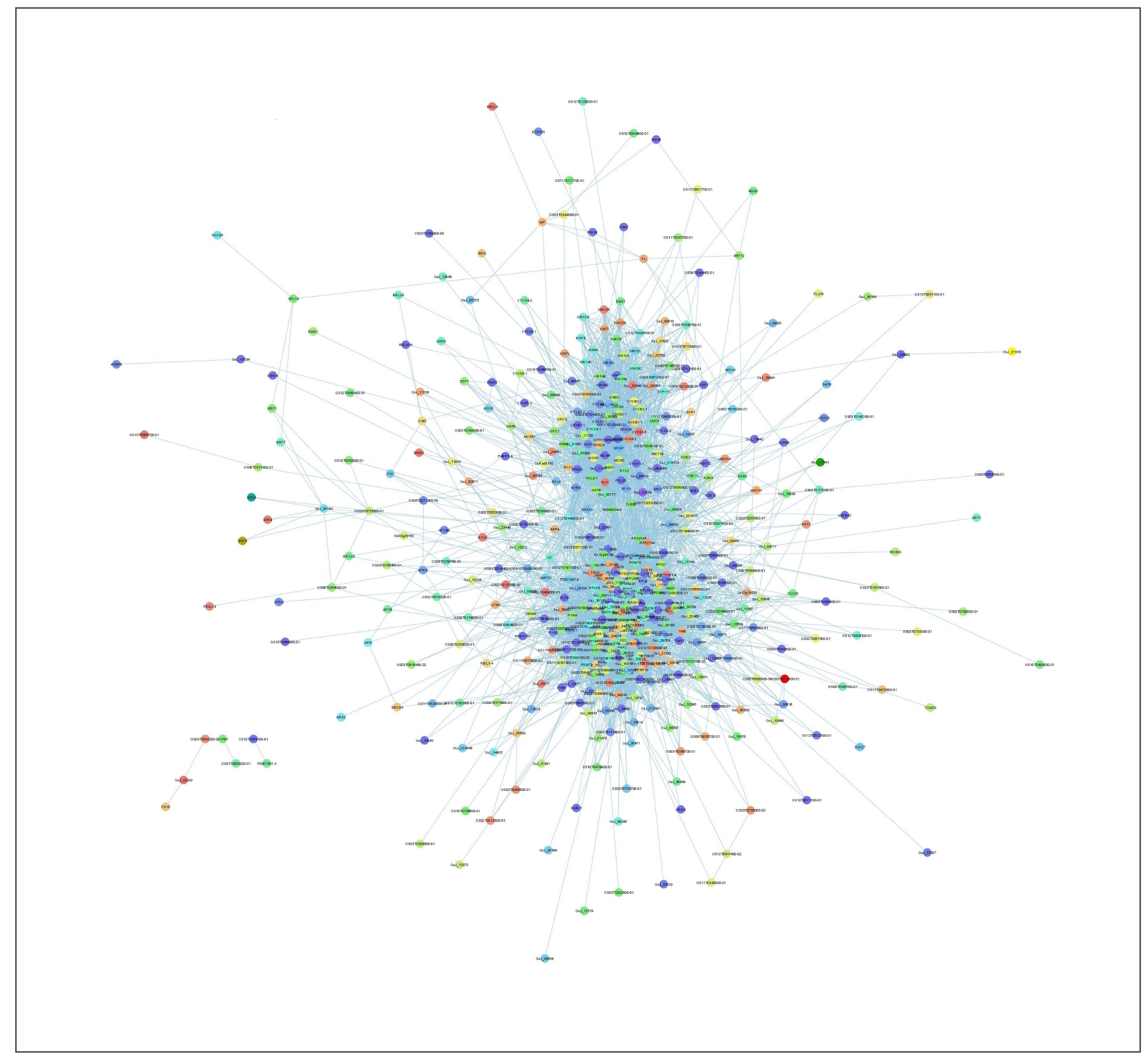


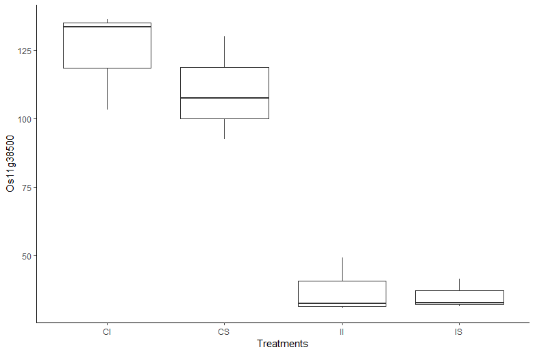


OsFBDUF62

Network of interaction protein-protein generated with genes from network Blue. Parameters of the network (String): number of nodes=549; number of edges=8238; average node degree=30; average local clustering coefficient=0.441; expected number of edges=4911; PPI enrichment *p*-value=<1.0e-16.


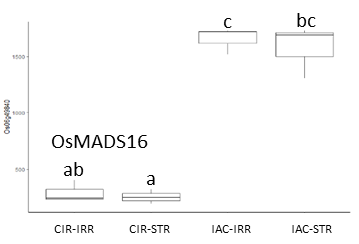

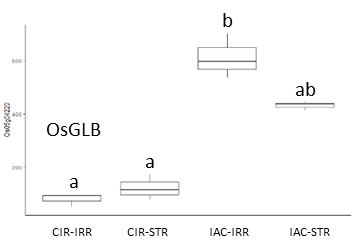

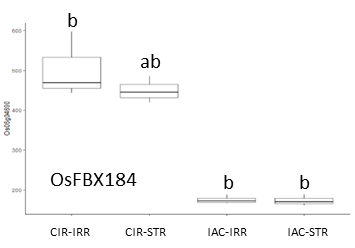

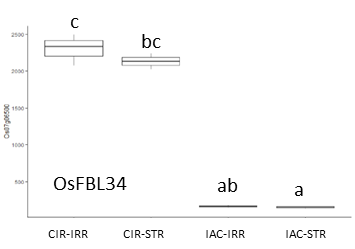

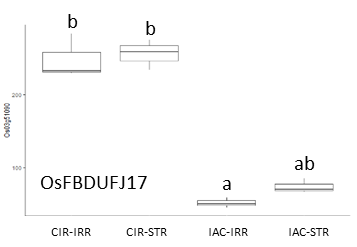


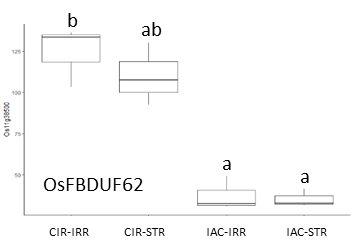


Box plot of core genes correlated to CIR-IRR. Comparison of variance was calculated using the Kruskall-Wallis and groups calculated using the Dunn test. Significance at p-value <0.05 is indicated by a different letter for genotype x treatments.


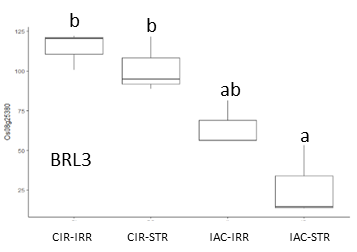

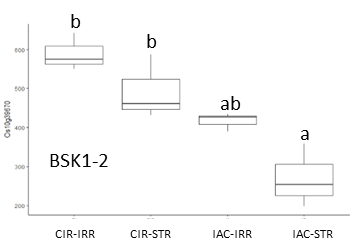


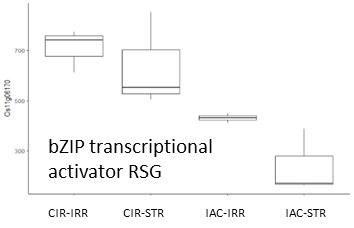

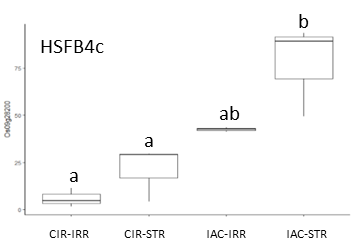

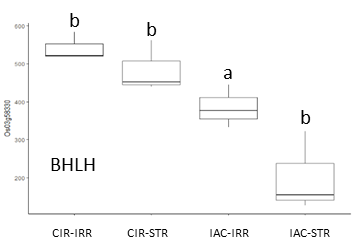


Box plot of core genes correlated to IAC-STR. Comparison of variance was calculated using the Kruskall-Wallis and groups calculated using the Dunn test. Significance at p-value <0.05 is indicated by a different letter for genotype x treatments.
