# Supplementary material for: A multi‐level approach reveals key physiological and molecular traits in the response of two rice genotypes subjected to water deficit at the reproductive stage
Source: Plant Environ Interact. 2023 Sep 15;4(5):229–57. doi: 10.1002/pei3.10121 (PMC10564380; doi:10.1002/pei3.10121)
Supplement: Supplementary file 4 — Table S1 [file PEI3-4-229-s004.docx]

**TABLE S1: List of the phenotypic traits measured**

| **Trait type** | **At the whole plant level** | | **On the main tiller** | |
| --- | --- | --- | --- | --- |
|  | **Phenotypic traits** | **Abbreviation** | **Phenotypic traits** | **Abbreviation** |
| **Duration** |  |  | Phyllochron | Phyllo_Repr |
| **Organogenesis** | Number of tillers | Tiller_N° | Number of panicle branches  Number of panicle spikelets | TotBranch_N°  Spiklt_N° |
| **Morphogenesis** | Shoot biomass (g)  Plant leaf area (mm²) Main tiller biomass (g) | Shoot_Biom  TotLeaf_Area MainTil_Biom | Plant height (mm)  Panicle biomass (g)  Panicle length (mm)  Flag leaf length (mm)  Flag leaf width (mm)  F-1 leaf length (mm)  F-1 leaf width (mm)  Internode1 length (mm)  Internode 1 diameter (mm)  Internode 2 length (mm)  Internode 2 diameter (mm)  Internode 3 length (mm)  Internode 3 diameter (mm)  Internode biomass (g)  Internode length (mm)  Peduncle length (mm)  Peduncle diameter (mm)  Total branch length (mm) | Plant_Height  Pan_Biom  Pan_Length  FlagLeaf_Length FlagLEaf_Width  F-1_Length  F-1_Width  IN1_Length  IN1_Diam  IN2_Length  IN2_Diam  IN3_Length  IN3_Diam  INTot_Biom  INTot_Length  Ped_Length  Ped_Diam  TotBranch_Length |
| **Sugar content** |  |  | Flag leaf hexose (mg/g)  Flag leaf sucrose (mg/g)  Flag leaf starch (mg/g)  Internode1 hexose (mg/g)  Internode1 sucrose (mg/g)  Internode1 starch (mg/g)  Internode2 hexose (mg/g)  Internode2 sucrose (mg/g)  Internode2 starch (mg/g) | FL_Hex  FL_Sucr  FL_Starch  IN1_Hex  IN1_Sucr  IN1_Starch  IN2_Hex  IN2_Sucr  IN2_Starch |
| **Physiological indicator** | Cumulative water use efficiency | CumWUE (mg DM g^-1^H_²_O) | Flag leaf specific leaf area (cm².g^-1^)  Flag leaf CO² assimilation rate (µmol CO² m^-2^s^-1^)  Flag leaf Assimilation rate per unit chlorophyll content (µmol CO² m^-2^s^-1^ spad unit^-1)^  Flag leaf internal CO² content (µmol CO² mol^-1^)  Flag leaf transpiration rate (mmol H²O m^-2^s^-1^)  Flag leaf instantaneous water use efficiency (µmol CO² mmol^-1^ H2O) | SLA  An  An/Spad  Ci  Tr  InstWUE, |
